# Birds on the move in the face of climate change: High species turnover in northern Europe

**DOI:** 10.1002/ece3.3328

**Published:** 2017-09-06

**Authors:** Raimo Virkkala, Aleksi Lehikoinen

**Affiliations:** ^1^ Finnish Environment Institute Natural Environment Centre Helsinki Finland; ^2^ Finnish Museum of Natural History University of Helsinki Helsinki Finland

**Keywords:** birds, climate change, land use, long‐distance migrants, range changes, species turnover

## Abstract

Species richness is predicted to increase in the northern latitudes in the warming climate due to ranges of many southern species expanding northwards. We studied changes in the composition of the whole avifauna and in bird species richness in a period of already warming climate in Finland (in northern Europe) covering 1,100 km in south–north gradient across the boreal zone (over 300,000 km^2^). We compared bird species richness and species‐specific changes (for all 235 bird species that occur in Finland) in range size (number of squares occupied) and range shifts (measured as median of area of occupancy) based on bird atlas studies between 1974–1989 and 2006–2010. In addition, we tested how the habitat preference and migration strategy of species explain species‐specific variation in the change of the range size. The study was carried out in 10 km squares with similar research intensity in both time periods. The species richness did not change significantly between the two time periods. The composition of the bird fauna, however, changed considerably with 37.0% of species showing an increase and 34.9% a decrease in the numbers of occupied squares, that is, about equal number of species gained and lost their range. Altogether 95.7% of all species (225/235) showed changes either in the numbers of occupied squares or they experienced a range shift (or both). The range size of archipelago birds increased and long‐distance migrants declined significantly. Range loss observed in long‐distance migrants is in line with the observed population declines of long‐distance migrants in the whole Europe. The results show that there is an ongoing considerable species turnover due to climate change and due to land use and other direct human influence. High bird species turnover observed in northern Europe may also affect the functional diversity of species communities.

## 1 INTRODUCTION

Global climate change is a major threat to biodiversity (Bellard, Bertelsmeier, Leadley, Thuiller, & Courchamp, 2012; Pereira et al., 2010), already having a considerable effect on species populations and communities (Chen, Hill, Ohleműller, Roy, & Thomas, 2011; Hickling, Roy, Hill, Fox, & Thomas, 2006; Parmesan, 2006; Stephens et al., 2016). Climate warming is projected to cause accelerating poleward and upward range shifts in different taxa (Barbet‐Massin, Thuiller, & Jiguet, 2012; Bellard et al., 2012). In Europe, bird species distributions are expected to change considerably in the 21st century due to climate change (Huntley, Collingham, Willis, & Green, 2008). The European‐wide bird monitoring data shows that species which have been predicted to gain range in the 21st century in Europe have increased, and those predicted to lose range declined between 1980 and 2005 (Gregory et al., 2009). Based on long‐term monitoring in Europe and North America, the responses of bird populations to climate change seem to be consistent between the two continents (Stephens et al., 2016).

Climate change is most probably the primary driver of the observed density changes and range shifts of species, but land use changes and other factors caused by humans are also highly important (see Clavero, Villero, & Brotons, 2011; Oliver et al., 2017). Climate change may rapidly affect the composition of bird communities by changing species’ relative abundances (Lindström, Green, Paulson, Smith, & Devictor, 2013). Population sizes of northern species are typically observed to be declining with ranges contracting while southern species are increasing in a given geographic region in northern latitudes (Virkkala & Rajasärkkä, 2011), and species ranges appear to be moving polewards (Brommer, Lehikoinen, & Valkama, 2012).

The potential future impacts of climate change on species distributions have commonly been assessed with bioclimatic envelope models (or species distribution or ecological niche models), whereby the relationships between present‐day distributions and climatic variables are modeled and then used to forecast the changes in a suitable climate space for species (Araújo & Peterson, 2012; Heikkinen, Luoto, Araújo, et al., 2006; Pearson & Dawson, 2003; Thuiller, Lafourcade, Engler, & Araújo, 2009). Barbet‐Massin et al. (2012) and Huntley, Green, Collingham, and Willis (2007) predicted the changes in bird species richness by 2050 and by the end of 21st century in the whole of Europe based on bioclimatic modeling of each individual species, and similarly, Thuiller et al. (2014) studied changes in functional diversity of European avian assemblages by 2080.

Here, we examined the observed changes in bird species’ distributions and numbers of squares occupied between two bird atlas (with 10 km squares) recording periods, the 1970s–1980s and 2006–2010 in Finland, covering 1,100 km in a south–north direction across the boreal zone in northern Europe. The study time span coincides with considerable warming of climate (see Table 1) which is also predicted to be much stronger in northern than in central and southern Europe in the 21st century. According to the Intergovernmental Panel on Climate Change (IPCC), the projected annual warming estimated by the A1B ensemble mean scenario for the end of 21st century is 5°C in the Arctic (including boreal areas), compared to 3.2–3.5°C in Europe in general (Christensen et al., 2007). From a global perspective, the boreal forest is the biome where climate is predicted to change rapidly. In a comparison of the world's 14 main biomes and their respective protected areas, Loarie et al. (2009) showed that climate residence time (i.e., the expected time for current climate to cross a given area) was among the lowest in protected areas of the boreal biome.

**Table 1:** Mean annual temperature (°C, *T*_Ann_), mean April–June temperature (°C, *T*_AMJ_), annual sum of growing degree days above 5°C (GDD5), and mean annual precipitation (in mm) in 1973–1979, 1985–1989, and 2005–2010

Climate variable	1973–1979	1985–1989	2005–2010
*T*_Ann_	1.65	1.24	2.88
*T*_AMJ_	6.47	6.74	7.51
GDD5	948	993	1,115
Precipitation	543	597	612

Furthermore, it is important to understand what species’ traits are important in explaining the variation in species‐specific responses and thus explain the general patterns. For example, among land birds, direction and speed of the density shifts have been influenced by migration strategy and habitat preference of species (Lehikoinen & Virkkala, 2016; Välimäki, Lindén, & Lehikoinen, 2016). In addition, long‐distance migrants have in general been declining in Europe compared to other migratory groups presumably due to changes in breeding areas, in wintering areas and in migration routes (e.g. Laaksonen & Lehikoinen, 2013; Sanderson, Donald, Pain, Burfield, & van Bommel, 2006). Importantly, in this study, we included all bird species including waterbirds that are often lacking in these analyses of functional groups.

We studied changes in bird species richness within an area of over 300,000 km^2^, within spatial square units sampled with similar research intensity between 1974 and 2010. Variation in observation effort may seriously jeopardize the results and conclusions of atlas studies, and therefore, it is highly important to take the research intensity into account (see Kujala, Vepsäläinen, Zuckerberg, & Brommer, 2013). We studied all bird species observed as breeders to look (1) at possible changes in species richness and composition in order to evaluate whether species richness has already increased in the northern latitudes as predicted by the bioclimatic modeling (Barbet‐Massin et al., 2012; Huntley et al., 2007). Virkkala, Pöyry, Heikkinen, Lehikoinen, and Valkama (2014) showed that ranges of northern bird species had already changed in the same direction as the predictions of species‐climate change models. Another essential question is (2) how many species have shifted their ranges by gaining or losing range and how these may differ between different orders, and how direction of shift differs between species gaining or losing range. (3) Moreover, we studied, whether there are any consistent patterns in the changes in species composition in relation to migratory status and habitat preference. Have resident versus migratory bird species gained or lost ranges and are there any patterns between different habitats with certain habitat including more species with expanding ranges and another including more species with contracting ranges?

## 2 METHODS

### 2.1 Bird atlases

We used data from three bird atlas studies carried out in Finland: Field work was carried out in 1974–1979, 1986–1989, and in 2006–2010 (Brommer et al., 2012; Hyytiä, Kellomäki, & Koistinen, 1983; Väisänen, Lammi, & Koskimies, 1998; Valkama, Vepsäläinen, & Lehikoinen, 2011). We pooled the information of the first two bird atlas surveys carried out in 1974–1979 and in 1986–1989 (Väisänen et al., 1998). This was performed because the third atlas in 2006–2010 was much more thorough (for categories of survey activity, see Väisänen (1989)) than the first two (Valkama et al., 2011), and atlas studies are susceptible to variations in survey effort (see Kujala et al., 2013). The second atlas was also partly concentrated on poorly studied regions of the first atlas (Väisänen et al., 1998), for which reason the first and second atlases were not comparable to the thorough third atlas. Surveys for the Finnish atlases were carried out using a uniform grid system of 10 × 10 km and the level of breeding status of bird species (recorded by bird observers), and survey activity (calculated based on number of species observations with varying breeding status included; Väisänen (1998); Väisänen et al. (1998)) in each square was recorded.

Pooling the first two atlases may cause problems in observing species with high year‐to‐year variation, such as bird species of prey depending on fluctuating vole populations. The probability for a population peak year for a bird species is thus higher in the first period with more study years than in the second period. However, the second period (2006–2010) coincided with an exceptionally high vole peak in 2008–2009 with high numbers of breeding vole‐eating birds of prey over large areas in Finland (Björklund, Honkala, & Saurola, 2009; Honkala, Björklund, & Saurola, 2010).

The breeding status of bird species recorded in each of the grid squares was assessed using four classes: 0 = not found, 1 = breeding possible (e.g., singing or displaying male observed once in a typical nesting habitat), 2 = breeding probable (e.g., singing or displaying male with a persistent territory observed, or female or pair present on more than one day in the same place, or bird observed building a nest), and 3 = confirmed breeding (Väisänen, 1989; Väisänen et al., 1998). For the analyses of this study, we combined classes 1, 2 and 3 to indicate species presence.

The atlas surveys graded the survey activity in each square according to six categories: 0 = no observations, 1 = occasional observations, 2 = fair surveys, 3 = satisfactory survey of the square, 4 = well‐surveyed, and 5 = thoroughly surveyed squares (Väisänen, 1989; Väisänen et al., 1998). This grading was developed during the second atlas (1986–1989) where observers (together with regional atlas organizer) evaluated their survey grade according to this classification, and the resulting sum of breeding status of species in each category was used as a basis to evaluate the survey grade in all the other atlases by taking into account the location (latitude) and land area (e.g., coastal areas) of each square (for details of the calculation, see Väisänen (1998)).

To control for the potential impacts of variation in survey efficiency, we only included squares with at least fair surveys (2–5) in both periods (1974–1989 and 2006–2010) and with exactly the same category of survey efficiency between the two periods. Originally, there were 3,813 grid squares covering the entire country, of which 1,622 squares (42.5%) fulfilled these survey effort requirements for the comparison. Because species numbers observed affects the survey activity measured, we took into account both squares with at least fair surveys (survey grade 2–5) and squares with thoroughly surveyed squares (survey grade 5) only in our analyses.

**Figure 1:**
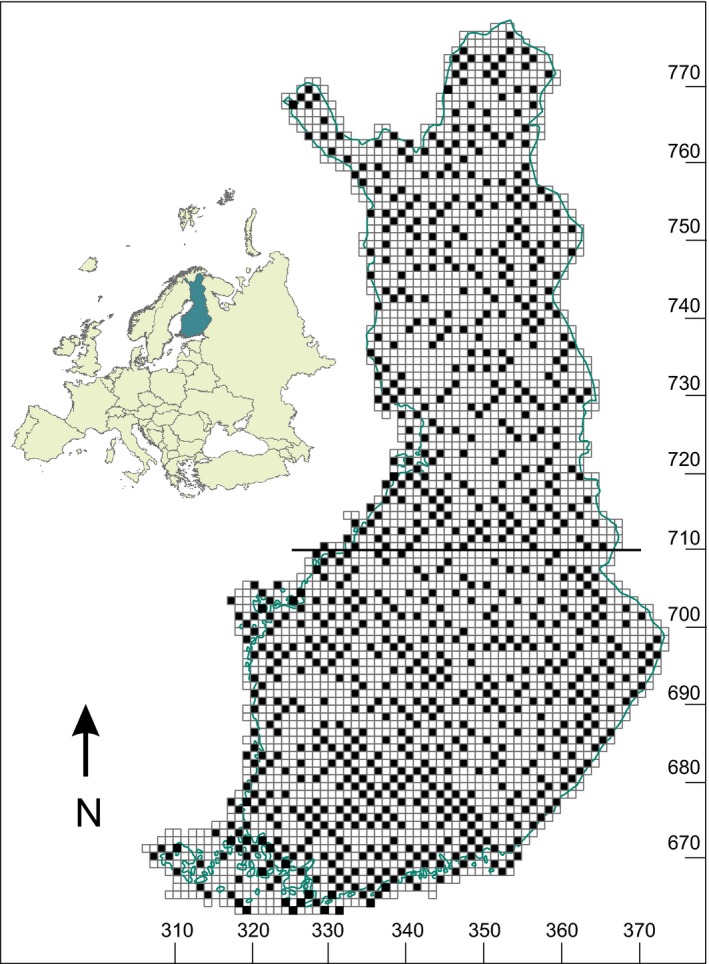
Location of the 10 × 10 km study squares in Finland based on a uniform grid. Bold line depicts the border between southern and northern Finland

However, atlas data sets often show high levels of spatial autocorrelation among grid squares situated geographically closely to each other (e.g., Dormann, 2007; Legendre, 1993) thus causing pseudoreplication in sampling. To avoid this, we excluded the closest, attached squares horizontally and vertically. This would for example reduce the overlap of 1‐km buffer zone of each square with other squares by about 93%, if all squares would be considered. Due to exclusion of adjacent squares, 848 of the 1,622 squares of similar survey grade were included in the sampling procedure in the analyses, which was 22.2% of all the squares (848/3,813; see Fig. 1).

### 2.2 Bird species

We took into account in the analyses all bird species observed in at least eight squares throughout the atlases (*N* = 235). We excluded only one species, the Arctic redpoll *Carduelis hornemanni*, because new knowledge of the identification of the Arctic redpoll emerged between the atlases so that bird observers could identify the species more reliably (Valkama et al., 2011). However, based on genetic analyses, the species status of the Arctic redpoll is not clear (Marthinsen, Wennerberg, & Lifjeld, 2008).

In the analyses, species were divided based on habitat preferences (five classes) and migratory status (four classes). Species were divided according to their habitat preferences as follows: (1) species of farmland and urban areas, (2) species of forest and scrubland, (3) species of wetlands and lakes, (4) species of Arctic mountain habitats, and (5) species of archipelago. Migratory status was recorded as (1) resident, (2) partial migrant, (3) short‐distance migrant, and (4) long‐distance migrant (Table S1; Väisänen et al., 1998; Laaksonen & Lehikoinen, 2013; Lehikoinen & Virkkala, 2016).

### 2.3 Statistical analyses

We used general linear models and generalized linear mixed models in our analyses. General linear models were used in analysing changes in species richness within squares. In this analysis, fixed effects were period (1974–1989 and 2006–2010) and region (southern and northern Finland, uniform grid 710 at south‐north axis as the border line, see Fig. 1) including the interaction term, and the dependent variable being species numbers in a square. The analysis was carried out both by including all squares with the same survey grade between the periods and by including thoroughly surveyed squares (research grade 5) only.

For studying the change in the numbers of occupied squares species‐specifically, we used a generalized linear mixed model (GLMM). In this model, species occurrence (present/absent) was studied using binomial distribution with logit function. Period (1974–1989 and 2006–2010) was regarded as a fixed effect and squares as a random effect. This analysis was carried out for 232 species.

For three species which were absent from the study squares either in the first or in the latter period, McNemar's test (a test for paired nominal data, see, e.g., Sokal & Rohlf, 1997) was used (great cormorant *Phalacrocorax carbo*, bearded tit *Panurus biarmicus*, and yellow‐breasted bunting *Emberiza aureola*) (Table S1). Great cormorant was not observed as a breeder in Finland in 1974–1989, bearded tit was observed in one square in 1974–1989, but not in the squares of the study, and yellow‐breasted bunting went extinct in Finland from 1974–1989 to 2006–2010 (the last observation in 2007, Valkama et al. (2011).

In studying location of a species range in the two time periods, we used median square both latitudinally and longitudinally and calculated the possible range shift (in km) between 1974–1989 and 2006–2010. This analysis shows the median location of area of occupancy between the two periods. Only species having occupied at least five squares in both the periods were included in this analysis (*N* = 226, see Table S1). In comparison of range shifts of different species groups, we used chi‐squared test (Chi‐square test for independence).

Furthermore, we studied what factors explain changes in species range size. In this analysis, we used range size of species in each atlas period as a response variable, which was explained by period (1974–1989 or 2006–2010), migration strategy (resident, partial migrant, short‐distance migrant, and long‐distance migrant; see Table S1), and habitat preference (farmland and urban areas, forest and scrubland, wetland and lakes, Arctic mountain habitats, and archipelago, Table S1). The interaction between period and migration strategy and period and habitat preference would test whether the range change between periods was dependent on these species traits. Species was a random factor in the model. As closely related species may have similar species traits due to common ancestor, we took phylogeny into account using phylogenic trees from www.birdtrees.org (Jetz, Thomas, Joy, Hartmann, & Mooers, 2012). The web‐site provides alternative trees (default 100 trees) for given species, and we selected the first ten of 100 trees for our analyses. The phylogeny was included in the model so that the closely related species had less weight in the analyses (inverse of tree). We used R‐package MCMCglmm, which conducts Bayesian generalized linear mixed models with Markov chain Monte Carlo methods (Hadfield, Nutall, Osorio, & Owens, 2007), to run the analyses (priors: list(R = list(V = 1, nu = 0.00), G = list(G1 = list(V = 1, nu = 0.02)))). We ran the full model using ten different trees and then ranked the model based on DIC‐value (similar to AIC; Burnham & Anderson, 2004). All the models were clearly within 2 ∆DIC (Table S2), which suggest that variation in the phylogenic tree does not make much difference. We thus selected the top ranked model (TREE9) for further analyses.

### 2.4 Climate data

We compared variation and trends of four climate variables known to be among the main climatic drivers affecting bird species distributions (e.g. Heikkinen, Luoto, & Virkkala, 2006; Huntley et al., 2007) mean annual temperature (*T*_Ann_), mean temperature of April–June (*T*_AMJ_), annual temperature sum above 5°C (growing degree days, GDD5), and annual precipitation (mm). These data for these climate variables are based on 10 × 10 km gridded data in Finland obtained from the Finnish Meteorological Institute (Tietäväinen, Tuomenvirta, & Venäläinen, 2010). We included the values of the climate variables in the study years and a year preceding each atlas study: 1973–1979, 1985–1989, and 2005–2010 (Table 1). Both mean annual temperature and mean April–June temperature rose over 1°C during the study period. In 1984/1985 and 1986/1987, there were exceptionally cold winters in the whole of central and northern Europe which is reflected in the low mean annual temperature in 1985–1989. Number of GDD5 has increased considerably, about 18% since the 1970s, as has annual precipitation (Table 1).

## 3 RESULTS

Bird species numbers per square (mean ± *SE*) was 94.71 ± 1.03 in 1974–1989 and 96.38 ± 1.08 in 2006–2010. Species numbers did not differ significantly between the two periods (Table 2), and the pattern was similar both in southern and northern Finland, because the interaction term between the periods and geographical regions was not significant. Apparently, species numbers in northern Finland were clearly lower than in southern Finland (*p* < .001). If only thoroughly surveyed squares were compared, species numbers per square were 114.02 ± 1.06 in 1974–1989 and 116.64 ± 1.11 in 2006–2010 (difference non‐significant, *p* = .069). Four hundred and forty‐eight of 848 squares were thoroughly surveyed but most of them (343) were in southern Finland.

**Table 2:** Comparison of species numbers per square between 1974–1989 and 2006–2010 based on general linear model with time periods and region (southern or northern Finland) as fixed factors in all squares (*df* = 1,692) (A) and in thoroughly surveyed squares only (*df* = 892) (B)

Source	A		B	
	*F*	*p*	*F*	*p*
Period	1.289	.256	3.309	.069
Region	740.506	<.001	73.099	<.001
Interaction	0.615	.433	0.358	.550

**Figure 2:**
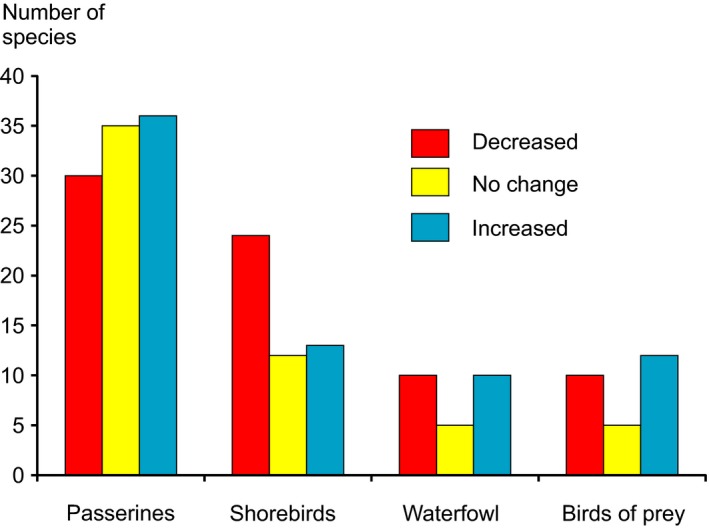
Numbers of species with decreased and those of species with increased numbers of occupied squares and species with no change in the numbers of occupied squares in the different bird orders: passerines (Passeriformes), shorebirds (Charadriiformes and Gruiformes), waterfowl (Anseriformes), and birds of prey (raptors Falconiformes and owls Strigiformes)

Based on species‐specific, generalized linear mixed model analyses (GLMM) with time as a fixed factor and square as a random factor, 87 species (37.0%) showed a significant (*p* < .05) increase in numbers of occupied squares and 82 species (34.9%) a decrease, and 66 species (28.1%) did not show any significant change (see Table S1). The numbers of decreased, stable, or increased species in terms of occupied squares did not significantly differ between major bird species orders (χ^2^ = 8.54, *p* = .201, *df* = 6), between passerines (Passeriformes), shorebirds (Charadriiformes and Gruiformes), waterfowl (Anseriformes), and birds of prey (raptors Falconiformes and owls Strigiformes) (Fig. 2).

**Figure 3:**
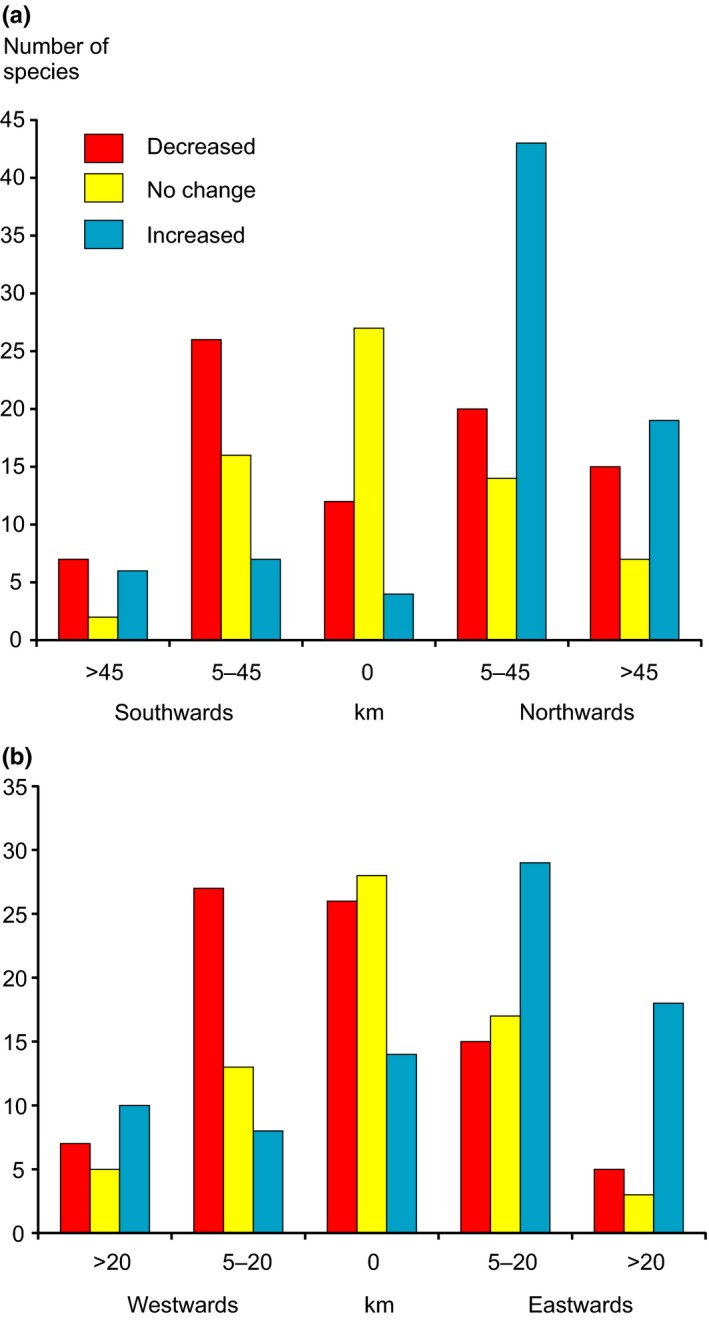
Latitudinal (a) and longitudinal (b) range shifts of decreased and increased species in terms of occupied squares and species with no change in occupied squares based on median square in 1974–1989 and in 2006–2010

The median range of over half of the species (52.7%) shifted at least 5 km northwards, while in the longitudinal gradient 38.5% of species moved eastwards and 31.4% westwards (Fig. 3). Patterns of both latitudinal and longitudinal shifts in median ranges between species having lost ranges, gained ranges, and species with no change in occupied squares differed significantly (latitudinal χ^2^ = 56.85, *p* < .001, *df* = 8; longitudinal: χ^2^ = 35.94, *p* < .001, *df* = 8). Median ranges of species with gained ranges moved the most, on average 23.88 km northwards and 8.88 km eastwards (20°), whereas those of species having lost ranges moved on average 13.56 km northwards and 3.25 km westwards (347°), and median ranges of species with no change in numbers of occupied squares moved 8.94 km northwards and 2.2 km westwards (346°).

The largest latitudinal changes in median ranges in the species losing range were in the dunlin *Calidris alpina* (265 km northwards in median range), brambling *Fringilla montifringilla* (170 km), Arctic warbler *Phylloscopus borealis* (170 km), hawk owl *Surnia ulula* (150 km), and willow ptarmigan *Lagopus lagopus* (145 km), which all are northern species. The largest changes in range size in the species gaining range were in the white‐tailed eagle *Haliaeetus albicilla* (265 km northwards in median range), Montagu's harrier *Circus pygargus* (260 km northwards), and whooper swan *Cygnus cygnus* (170 km southwards) (see Table S1).

The majority of the species that declined were indeed northern, but numbers of occupied squares have also declined in certain southern agricultural species for which ranges retreated southwards (e.g., common starling *Sturnus vulgaris*, Eurasian skylark *Alauda arvensi*s, and ortolan bunting *Emberiza hortulana*) as also in certain southern waterbirds of wetlands and lakes (e.g., common pochard *Aythya ferina* and great crested grebe *Podiceps cristatus*) (see Table S1).

Although the majority of the threatened and near threatened species (Tiainen et al., 2016) have declined, many red‐listed species, such as white‐tailed eagle, golden eagle *Aquila chrysaetos*, peregrine *Falco peregrinus*, and white‐backed woodpecker *Dendrocopos leucotos* have increased their ranges between 1974–1989 and 2006–2010 (Table S1).

There were only 10 species which did not show both any significant change in the numbers of occupied squares and shift in median ranges (neither latitudinally nor longitudinally) at all between the study periods. Seven of these species were among the 20 most abundant species in Finland according to Väisänen et al. (1998) and mostly distributed over the whole country: willow warbler *Phylloscopus trochilus*, common chaffinch *Fringilla coelebs*, tree pipit *Anthus trivialis*, redwing *Turdus iliacus*, spotted flycatcher *Muscicapa striata*, common redstart *Phoenicurus phoenicurus*, and garden warbler *Sylvia borin*. Thus, there were changes either in the numbers of occupied squares or in range shifts (or in both), in 95.7% of all species (225/235).

Both migration strategy and habitat preference explained part of the variation in the species‐specific range size between the two atlas periods when phylogeny was taken into account (Table 3). Significant interaction between long‐distance migrants and period suggested that long‐distance migrants showed reduced range size compared to residents, but other migratory groups did not show a similar pattern (Table 3). In addition, significant interactions suggested that archipelago birds have increased their range size compared to farmland birds, and there was a tendency that range size of mountain birds was reduced compared to farmland species. Range sizes of species preferring other habitats (forest and wetland species) did not show any difference compared to farmland species (Table 3).

**Table 3:** Parameter estimates (posterior mean including min–max values) and *p*‐value based on the model explaining species‐specific variation in range size change. Period is atlas period (1974–1989 or 2006–2010). Mig is migration behavior (partial, short‐distance, SDM, and long‐distance migrants, and LDM are compared to residents). Hab is habitat preference (forest, wetland, mountain, and archipelago species are compared to farmland species). Significant coefficients are bolded

Variable	Post. mean [min, max]	*p*
(Intercept)	5.66 [3.12, 8.34]	<.001
Period	0.24 [0.02, 0.50]	.051
Mig (Partial)	0.35 [−0.57, 1.17]	.434
Mig (SDM)	0.12 [−0.72, 0.99]	.798
Mig (LDM)	−0.62 [−1.42,0.33]	.197
Hab (Forest)	0.51 [−0.19, 1.22]	.174
Hab (Wetland)	−0.86 [−1.75, −0.12]	.051
Hab (Mountain)	**−2.33 [−3.18, −1.46]**	**<.001**
Hab (Archipelago)	**−2.23 [−3.10, −1.30]**	**<.001**
Period * Partial	−0.14 [−0.41, 0.12]	.349
Period * SDM	−0.16 [−0.37, 0.07]	.194
Period * LDM	**−0.37 [−0.61,−0.15]**	**.002**
Period * Forest	0.01 [−0.19, 0.23]	.945
Period * Wetland	0.14 [−0.07, 0.36]	.223
Period * Mountain	−0.32 [−0.62, 0.05]	.069
Period * Archipelago	**0.31 [0.02, 0.59]**	**.036**

## 4 DISCUSSION

The significance of climate change affecting species distributions and population changes has been increasingly emphasized during the past decades (Bellard et al., 2012; Pereira et al., 2010; Sala et al., 2000; Scheffers et al., 2016). The present data are consistent with range shifts caused by climate warming in northern Europe, but there is no increase in bird species richness, at least not at the scale of 10 × 10 km. In contrast, species turnover seems to be very high with considerable range shifts of species, and numbers of species gaining range, and species losing range being about equal. Thus, the prediction of increasing species richness with climate warming in the northern latitudes seems not to have occurred so far, and it seems that ranges of many northern species retreat as southern species expand (Brommer et al., 2012). Interestingly, ranges of almost all species have changed, and range shifts occurred in different directions both latitudinally and longitudinally (see also Gillings, Balmer, & Fuller, 2015), which was also observed in shifts of mean weighted population densities (Lehikoinen & Virkkala, 2016).

Although we did not find any change in species richness, and tried to standardize the possible differences in survey effort between the atlases and reduce the effects of spatial autocorrelation, there still is the possibility that species richness is overestimated in the last atlas compared with the earlier atlases, because the third atlas in 2006–2010 was more thorough than the pooled 1974–1989 atlases. For example, Tingley and Beissinger (2013) observed that avian richness declined in montane areas in western USA over a period of over 100 years but only after careful incorporation of changes in species detectability. Our interpretation in the present work is that species richness has not increased, but it remains possible that richness might have declined but, however, the present method used could not detect such changes.

In our work, we observed that species turnover is high, which also means that structure of bird communities is changing and totally new community composition may evolve. Interestingly, records from Glacial period in Northern England (55–40 kyr before present, Stewart & Jacobi, 2015) showed that there were both northern (such as willow ptarmigan/red grouse *Lagopus lagopus,* and rock ptarmigan *L. muta*), southern alpine (such as Alpine swift *Tachymarptis melba*), and eastern steppe bird species present (such as demoiselle crane *Grus virgo* and long‐legged buzzard *Buteo rufinus*) at the same site. Currently, these species do not have overlapping ranges, thus these ancient bird communities were nonanalogous to any present bird communities. Also in the future, novel climatic conditions will probably cause no‐analog bird communities to emerge (Stralberg et al., 2009; Wiens, Seavy, & Jongsomjit, 2011).

Numbers of species gaining or losing range do not greatly differ between habitats when phylogeny is taken into account. This means that the high bird species turnover occurs across various habitat types. There is a slightly negative pattern in range size in the birds of Arctic mountains which include northern species and a positive pattern in the birds of archipelago. Several species, many of which are southern and large in size, have increased considerably and expanded in the archipelago including mute swan *Cygnus olor*, greylag goose *Anser anser*, barnacle goose *Branta leucopsis*, common shelduck *Tadorna tadorna*, great cormorant, and white‐tailed eagle.

Large red‐listed bird species have increased as a consequence of conservation actions, decrease in pesticides (particularly birds of prey), and decrease in persecution. At the European scale, particularly large and rare species have increased largely due to conservation actions while the more common and smaller species have declined considerably (Inger et al., 2015). However, for most species, climate change and land use probably are the key factors affecting population change and range shift patterns (Fraixedas, Lindén, & Lehikoinen, 2015; Virkkala, 2016; Virkkala & Lehikoinen, 2014).

Clearly, the largest change was the significant range loss of long‐distance (tropical) migrants. Decline of long‐distance migrant populations has been observed both in Finland (Laaksonen & Lehikoinen, 2013) and in the whole of Europe during the past decades (Gregory et al., 2007; Sanderson et al., 2006). Changes in range sizes in birds are thus closely connected with changes in population abundance.

Barbet‐Massin et al. (2012) predicted the distribution of 409 European bird species based on bioclimatic modeling and observed that the range of 71% of all species would decrease by 2050. However, species richness was predicted to increase to a large extent only in northern Finland and Sweden and in northern Russia (in addition smaller areas in Iceland, in parts of Great Britain, northwestern Iberia, northern Turkey, and the Alps). Also, Huntley et al. (2007) predicted, based on their bioclimatic model, that bird species richness would increase considerably in Finland by 2070–2099 and the richness in Finland and in the neighboring areas (parts of Sweden and northwestern Russia) being the very highest in Europe at that time. However, it may well be that the predicted increase in species richness in the northern latitudes will not be as high as forecasted, and thus, the overall decline of bird species in Europe may be even higher than that modeled by Barbet‐Massin et al. (2012).

Virkkala et al. (2014) showed that range changes of birds between 1974–1989 and 2006–2010 in Finland are in the same direction as the predictions of bioclimatic models of the same species by 2051–2080 (Virkkala, Heikkinen, Fronzek, & Leikola, 2013). Therefore, one would expect that species richness would already have increased in Finland between 1974–1989 and 2006–2010. One reason for the discrepancy might be that there is a time lag in the effects of climate change on species (Devictor et al., 2012). In Finland, the temperature moved on average 186 km north north‐east between 1970–1989 and 2000–2012, but mean weighted density of 128 land bird species moved only 37 km north north‐east, on average, with high between‐species variation in the direction of density shifts (Lehikoinen & Virkkala, 2016).

On the other hand, the decline of also many southern species suggests that many species may not track climate change due to lack of suitable habitat out (north) of their present range. For example, the skylark and many other species of agricultural areas cannot move northwards because these species breed almost exclusively on fields, and there are no suitable agricultural areas for the species in the northernmost Finland. Dispersal barriers and lack of suitable habitat in the otherwise climatically suitable new areas may considerably restrict species range shifts. For range shifts of northern‐boreal and arctic species Arctic Ocean forms, almost an impenetrable dispersal barrier probably causing the local extinction of many of these species in northern continental Europe in the 21st century (Virkkala, Heikkinen, Leikola, & Luoto, 2008). Moreover, intensive agriculture (Laaksonen & Lehikoinen, 2013; Vepsäläinen, Tiainen, Holopainen, Piha, & Seimola, 2010) and forestry (Fraixedas et al., 2015; Virkkala, 2016) may negatively affect species occurring in these habitats and thus reducing the potential species richness. Thus, climate change is not the only driver affecting range and density shifts of species but intensive land use may cause degradation of habitats restricting species shifts (see also Lehikoinen & Virkkala, 2016).

High species turnover would also mean that functional diversity of species communities would change. Thuiller et al. (2014) studied changes in functional diversity of bird species groups by 2080 at the scale of whole of Europe based on bioclimatic envelope modeling. They observed that in northern Fennoscandia (including Finland), there was a decrease in functional diversity in the avian biota which was parallel to the predicted high regional turnover of individual species resulting in substantial changes in trophic relationships. It seems that observed patterns of high bird species turnover in northern Europe give support to the prediction of possible changes in functional diversity in avian biota which would have considerable effects on ecosystem services as a whole. Moreover, high bird species turnover affects between‐species relations and may ultimately have cascading effects on the bird communities (Auer & Martin, 2013; Zarnetske, Skelly, & Urban, 2012).

## AUTHOR CONTRIBUTIONS

The original idea was developed and design planned by RV. Both authors, RV and AL, contributed to methods, analyses, and writing.

## CONFLICT OF INTEREST

None declared.
