# Supplementary material for: Birds on the move in the face of climate change: High species turnover in northern Europe
Source: Ecol Evol. 2017 Sep 6;7(20):8201–9. doi: 10.1002/ece3.3328 (PMC5648647; doi:10.1002/ece3.3328)
Supplement: Supplementary file 1 [file ECE3-7-8201-s001.docx]

| Supplementary Table S1. Change in occupied squares of species between 1974–89 and 2006–10. 1 = increase, 2 = decrease, 3 = no change. Latitudinal (N-coord) and longitudinal (E-coord) median square in 1974–89 (Md 74–89) and 2006–10 (Md 2006–10) for each species (for calculation, see text). Values of F, df_2_ and significance level (P) in GLMM in each species. Asterisk denotes values of test statistic, N and significance level (p) in species analyzed by McNemar's test. Habitat: 1 = farmland and urban areas, 2 = forest and scrubland, 3 = wetland and lakes, 4 = Arctic mountain, 5 = archipelago. Migration: 1 = resident, 2 = partial migrant, 3 = short-distance migrant, 4 = long-distance migrant. | | | | | | | | | | |
| --- | --- | --- | --- | --- | --- | --- | --- | --- | --- | --- |
| Species | Change | Md 74–89. N-coord | Md  74–89. E-coord | Md 2006–10. N-coord | Md 2006–10. E-coord | F | df_2_/N | P | Hab. | Migr. |
| *Cygnus olor* | 1 | 670 | 321 | 672 | 328 | 48.136 | 298 | <0.001 | 5 | 2 |
| *Cygnus cygnus* | 1 | 716 | 347 | 699 | 344 | 323.231 | 1474 | <0.001 | 3 | 3 |
| *Anser fabalis* | 3 | 733 | 351 | 731.5 | 350 | 0.26 | 438 | 0.61 | 3 | 3 |
| *Anser anser* | 1 | 672 | 321 | 677 | 326 | 50.618 | 262 | <0.001 | 5 | 3 |
| *Branta canadensis* | 1 | 677.5 | 331 | 680 | 338 | 170.843 | 514 | <0.001 | 3 | 3 |
| *Branta leucopsis* | 1 | - | - | 677 | 336 | 64.103 | 166 | <0.001 | 5 | 3 |
| *Tadorna tadorna* | 1 | 678 | 320 | 680 | 326 | 13.967 | 92 | <0.001 | 5 | 3 |
| *Anas penelope* | 2 | 697 | 343 | 696 | 343 | 16.777 | 1394 | <0.001 | 3 | 3 |
| *Anas strepera* | 1 | 675 | 338 | 680 | 337 | 85.024 | 250 | <0.001 | 3 | 3 |
| *Anas crecca* | 3 | 699 | 344 | 698 | 344 | 0.006 | 1622 | 0.938 | 3 | 3 |
| *Anas platyrhynchos* | 3 | 696 | 343 | 696 | 342 | 1.731 | 1596 | 0.188 | 3 | 2 |
| *Anas acuta* | 2 | 700 | 342 | 702 | 342 | 115.296 | 948 | <0.001 | 3 | 3 |
| *Anas querquedula* | 2 | 685 | 340 | 689 | 340 | 49.142 | 582 | <0.001 | 3 | 4 |
| *Anas clypeata* | 2 | 687 | 337 | 689 | 338 | 7.395 | 792 | 0.007 | 3 | 3 |
| *Aythya ferina* | 2 | 682 | 338 | 680 | 338 | 60.404 | 604 | <0.001 | 3 | 3 |
| *Aythya fuligula* | 2 | 697 | 342 | 697 | 342 | 22.276 | 1410 | <0.001 | 3 | 3 |
| *Aythya marila* | 2 | 705 | 328 | 700 | 327 | 15.256 | 178 | <0.001 | 5 | 3 |
| *Somateria mollissima* | 3 | 670.5 | 321 | 671 | 321 | 0 | 196 | 1 | 5 | 3 |
| *Clangula hyemalis* | 2 | 763 | 342.5 | 765 | 340.5 | 9.011 | 122 | 0.003 | 4 | 3 |
| *Melanitta nigra* | 1 | 759 | 348 | 748 | 345 | 6.16 | 222 | 0.014 | 3 | 3 |
| *Melanitta fusca* | 2 | 702.5 | 332 | 703 | 330 | 7.838 | 342 | 0.005 | 5 | 3 |
| *Bucephala clangula* | 3 | 698 | 344 | 698 | 343.5 | 0.48 | 1638 | 0.488 | 3 | 3 |
| *Mergellus albellus* | 1 | 738.5 | 351 | 729 | 348 | 56.376 | 368 | <0.001 | 3 | 3 |
| *Mergus serrator* | 2 | 694 | 343 | 693 | 343 | 13.183 | 1172 | <0.001 | 3 | 3 |
| *Mergus merganser* | 1 | 692 | 342 | 693 | 343 | 12.36 | 1228 | <0.001 | 3 | 3 |
| *Tetrastes bonasia* | 3 | 694 | 343 | 695 | 344 | 0.028 | 1396 | 0.868 | 2 | 1 |
| *Lagopus lagopus* | 2 | 716 | 346 | 730.5 | 347 | 129.04 | 1060 | <0.001 | 2 | 1 |
| *Lagopus muta* | 3 | 767 | 347 | 766.5 | 347.5 | 0.298 | 70 | 0.587 | 4 | 1 |
| *Lyrurus tetrix* | 3 | 697 | 343 | 697 | 344 | 0 | 1530 | 1 | 2 | 1 |
| *Tetrao urogallus* | 2 | 698 | 344 | 700.5 | 345 | 29.105 | 1498 | <0.001 | 2 | 1 |
| *Perdix perdix* | 3 | 683.5 | 334.5 | 693 | 331 | 0.42 | 376 | 0.518 | 1 | 1 |
| *Coturnix coturnix* | 1 | 678 | 337 | 682 | 336 | 99.975 | 270 | <0.001 | 1 | 4 |
| *Phasianus colchicus* | 1 | 679 | 335 | 685.5 | 336 | 32.648 | 690 | <0.001 | 1 | 1 |
| *Gavia stellata* | 1 | 698 | 343 | 696 | 345 | 58.331 | 610 | <0.001 | 3 | 3 |
| *Gavia arctica* | 1 | 695 | 345 | 696 | 346 | 22.142 | 1184 | <0.001 | 3 | 3 |
| *Tachybaptus ruficollis* | 3 | 673 | 335.5 | 682 | 332 | 0.135 | 28 | 0.716 | 3 | 3 |
| *Podiceps cristatus* | 2 | 687 | 342 | 685 | 342 | 14.843 | 928 | <0.001 | 3 | 3 |
| *Podiceps grisegena* | 1 | 682.5 | 342 | 686 | 344 | 73.187 | 718 | <0.001 | 3 | 3 |
| *Podiceps auritus* | 2 | 685 | 336 | 686 | 336 | 3.981 | 614 | 0.046 | 3 | 3 |
| *Phalacrocorax carbo** | 1 | - | - | 674 | 326 | 67.014 | 69 | <0.001 | 5 | 3 |
| *Botaurus stellaris* | 1 | 675.5 | 342 | 682 | 342 | 121.31 | 466 | <0.001 | 3 | 3 |
| *Ardea cinerea* | 1 | 670 | 335 | 675 | 335 | 104.97 | 314 | <0.001 | 3 | 3 |
| *Pernis apivorus* | 2 | 689 | 342 | 689 | 342 | 10.726 | 1054 | 0.001 | 2 | 4 |
| *Milvus migrans* | 3 | 679 | 348 | 717 | 344 | 0.067 | 58 | 0.797 | 2 | 4 |
| *Haliaeetus albicilla* | 1 | 676 | 321 | 702.5 | 336 | 117.6 | 302 | <0.001 | 5 | 2 |
| *Circus aeruginosus* | 1 | 678.5 | 336 | 686 | 341 | 134.921 | 658 | <0.001 | 3 | 4 |
| *Circus cyaneus* | 2 | 707 | 344 | 701.5 | 344 | 28.467 | 920 | <0.001 | 3 | 3 |
| *Circus pygargus* | 1 | 670 | 336.5 | 696 | 329.5 | 5.989 | 38 | 0.019 | 3 | 4 |
| *Accipiter gentilis* | 2 | 693 | 342 | 693 | 342 | 8.484 | 1294 | 0.004 | 2 | 2 |
| *Accipiter nisus* | 3 | 692 | 341 | 692 | 342 | 0.666 | 1274 | 0.415 | 2 | 3 |
| *Buteo buteo* | 2 | 693 | 343 | 690 | 342 | 44.387 | 1262 | <0.001 | 2 | 3 |
| *Buteo lagopus* | 3 | 751 | 346 | 749 | 348 | 3.374 | 476 | 0.067 | 2 | 3 |
| *Aquila chrysaetos* | 1 | 742 | 348 | 749 | 348 | 16.308 | 362 | <0.001 | 2 | 2 |
| *Pandion haliaetus* | 1 | 690 | 343 | 692 | 344 | 18.408 | 1124 | <0.001 | 2 | 4 |
| *Falco tinnunculus* | 1 | 695 | 342 | 697 | 344 | 17.194 | 1396 | <0.001 | 1 | 3 |
| *Falco columbarius* | 2 | 709 | 345 | 708.5 | 346 | 6.611 | 942 | 0.01 | 2 | 3 |
| *Falco subbuteo* | 1 | 689 | 342 | 690 | 342 | 11.335 | 1168 | 0.001 | 2 | 4 |
| *Falco rusticolus* | 1 | 767 | 340 | 769 | 347 | 4.435 | 54 | 0.04 | 4 | 1 |
| *Falco peregrinus* | 1 | 745.5 | 348 | 745 | 347 | 23.261 | 224 | <0.001 | 3 | 3 |
| *Rallus aquaticus* | 1 | 676 | 335 | 679 | 334.5 | 57.855 | 350 | <0.001 | 3 | 3 |
| *Porzana porzana* | 2 | 681 | 341.5 | 682 | 340 | 19.423 | 544 | <0.001 | 3 | 4 |
| *Porzana parva* | 1 | 679.5 | 339 | 679 | 345 | 4.295 | 30 | 0.047 | 3 | 4 |
| *Crex crex* | 1 | 681 | 340 | 687 | 342 | 163.56 | 838 | <0.001 | 1 | 4 |
| *Gallinula chloropus* | 3 | 677 | 340 | 677 | 338 | 0 | 172 | 1 | 3 | 3 |
| *Fulica atra* | 2 | 681 | 337 | 681 | 337 | 14.421 | 652 | <0.001 | 3 | 3 |
| *Grus grus* | 1 | 697 | 344 | 697 | 344 | 65.025 | 1460 | <0.001 | 3 | 3 |
| *Haematopus ostralegus* | 1 | 678 | 329 | 683 | 333 | 51.684 | 444 | <0.001 | 5 | 3 |
| *Charadrius dubius* | 2 | 690 | 341 | 693 | 339 | 73.896 | 816 | <0.001 | 1 | 4 |
| *Charadrius hiaticula* | 2 | 723 | 339 | 723.5 | 337 | 25.028 | 496 | <0.001 | 4 | 3 |
| *Charadrius morinellus* | 3 | 767 | 347 | 765 | 347 | 1.242 | 50 | 0.27 | 4 | 3 |
| *Pluvialis apricaria* | 2 | 720 | 345 | 721 | 345 | 12.415 | 862 | <0.001 | 4 | 3 |
| *Vanellus vanellus* | 2 | 695 | 342 | 692 | 342 | 44.513 | 1332 | <0.001 | 1 | 3 |
| *Calidris temminckii* | 2 | 753 | 340 | 746 | 340 | 13.788 | 166 | <0.001 | 4 | 4 |
| *Calidris alpina* | 2 | 720 | 335 | 746.5 | 340 | 8.002 | 118 | 0.005 | 4 | 3 |
| *Calidris pugnax* | 2 | 720 | 345 | 719.5 | 341.5 | 178.07 | 778 | <0.001 | 3 | 4 |
| *Calidris falcinellus* | 2 | 746 | 350 | 752 | 349 | 9.797 | 208 | 0.002 | 3 | 4 |
| *Lymnocryptes minumus* | 2 | 739 | 348 | 737.5 | 348 | 13.28 | 392 | <0.001 | 3 | 3 |
| *Gallinago gallinago* | 2 | 700 | 344 | 698 | 344 | 13.023 | 1608 | <0.001 | 3 | 3 |
| *Gallinago media* | 1 | - | - | 683 | 341 | 7.401 | 20 | 0.013 | 3 | 4 |
| *Scolopax rusticola* | 1 | 690 | 341 | 693 | 342 | 13.169 | 1308 | <0.001 | 2 | 3 |
| *Limosa limosa* | 1 | 692.5 | 339.5 | 700 | 341 | 12.767 | 44 | 0.001 | 3 | 4 |
| *Limosa lapponica* | 3 | 770.5 | 349 | 770 | 349 | 1.299 | 66 | 0.259 | 4 | 4 |
| *Numenius phaeopus* | 3 | 730.5 | 348 | 727 | 348 | 2.54 | 832 | 0.111 | 3 | 4 |
| *Numenius arquata* | 2 | 695 | 342 | 695 | 343 | 9.688 | 1360 | 0.002 | 1 | 3 |
| *Tringa erythropus* | 2 | 748 | 349 | 749 | 349 | 15.931 | 432 | <0.001 | 3 | 4 |
| *Tringa totanus* | 2 | 685 | 333 | 684 | 335 | 5.763 | 672 | 0.017 | 5 | 3 |
| *Tringa stagnatilis* | 3 | 695 | 360 | 692 | 342 | 2.695 | 34 | 0.11 | 3 | 4 |
| *Tringa nebularia* | 3 | 713 | 349 | 711 | 348 | 0.153 | 1226 | 0.696 | 3 | 4 |
| *Tringa ochropus* | 1 | 693 | 342 | 695 | 343 | 14.021 | 1360 | <0.001 | 2 | 4 |
| *Tringa glareola* | 2 | 706 | 345 | 713 | 345 | 69.985 | 1430 | <0.001 | 3 | 4 |
| *Actitis hypoleucos* | 2 | 699 | 344 | 698 | 343 | 12.583 | 1656 | <0.001 | 3 | 4 |
| *Arenaria interpres* | 2 | 679.5 | 325 | 677 | 324 | 15.385 | 218 | <0.001 | 5 | 4 |
| *Phalaropus lobatus* | 2 | 749 | 348 | 756.5 | 347 | 71.462 | 304 | <0.001 | 3 | 4 |
| *Stercorarius parasiticus* | 1 | 680 | 320 | 683 | 320 | 7.674 | 150 | 0.006 | 5 | 4 |
| *Stercorarius longicaudus* | 2 | 767 | 346 | 768 | 346 | 4.467 | 88 | 0.037 | 4 | 4 |
| *Hydrocoloeus minutus* | 1 | 692 | 344 | 696 | 343 | 126.16 | 842 | <0.001 | 3 | 3 |
| *Chroicocephalus ridibundus* | 2 | 696 | 342 | 692 | 341 | 49.975 | 1424 | <0.001 | 3 | 3 |
| *Larus canus* | 1 | 694 | 342 | 696 | 342 | 15.331 | 1514 | <0.001 | 3 | 3 |
| *Larus fuscus* | 2 | 689 | 343 | 689 | 343 | 21.88 | 1084 | <0.001 | 3 | 4 |
| *Larus argentatus* | 1 | 690 | 341 | 692.5 | 342 | 8.942 | 1312 | 0.003 | 5 | 2 |
| *Larus marinus* | 3 | 678 | 327.5 | 678 | 329.5 | 3.073 | 340 | 0.08 | 5 | 2 |
| *Hydroprogne caspia* | 3 | 674 | 326 | 677 | 329 | 1.696 | 204 | 0.194 | 5 | 4 |
| *Sterna hirundo* | 3 | 692 | 342 | 692 | 342 | 0.183 | 1310 | 0.669 | 3 | 4 |
| *Sterna paradisaea* | 2 | 729 | 340 | 716 | 338 | 13.407 | 724 | <0.001 | 5 | 4 |
| *Sternula albifrons* | 3 | 718 | 338 | 718.5 | 338 | 0.983 | 40 | 0.327 | 5 | 4 |
| *Chlidonias niger* | 2 | 678 | 337.5 | 690 | 330 | 23.79 | 100 | <0.001 | 3 | 4 |
| *Alca torda* | 3 | 671 | 320 | 671 | 322 | 0.72 | 114 | 0.398 | 5 | 3 |
| *Cepphus grylle* | 3 | 672 | 320.5 | 671 | 320.5 | 3.15 | 160 | 0.078 | 5 | 3 |
| *Columba livia* | 3 | 682 | 336 | 682 | 338 | 0.011 | 546 | 0.916 | 1 | 1 |
| *Columba oenas* | 3 | 676 | 333 | 676 | 333 | 0.18 | 524 | 0.672 | 1 | 3 |
| *Columba palumbus* | 1 | 693 | 342 | 695 | 343 | 11.414 | 1390 | 0.001 | 1 | 3 |
| *Streptopelia decaocto* | 1 | 678.5 | 331.5 | 678 | 334.5 | 11.789 | 194 | 0.001 | 1 | 1 |
| *Streptopelia turtur* | 2 | 677.5 | 342.5 | 683.5 | 351.5 | 68.431 | 164 | <0.001 | 1 | 4 |
| *Cuculus canorus* | 3 | 699 | 344 | 699.5 | 344 | 0.054 | 1626 | 0.817 | 2 | 4 |
| *Bubo bubo* | 1 | 684 | 335 | 686 | 337 | 4.03 | 888 | 0.045 | 2 | 1 |
| *Bubo scandiacus* | 2 | 769 | 329 | - | - | 4.868 | 16 | 0.042 | 4 | 2 |
| *Surnia ulula* | 2 | 724 | 347 | 739 | 350 | 54.515 | 588 | <0.001 | 2 | 2 |
| *Glaucidium passerinum* | 1 | 685 | 337 | 688 | 339 | 174.75 | 850 | <0.001 | 2 | 1 |
| *Strix aluco* | 2 | 679 | 339 | 678 | 337 | 22.399 | 610 | <0.001 | 2 | 1 |
| *Strix uralensis* | 1 | 689 | 342 | 690 | 342.5 | 37.66 | 930 | <0.001 | 2 | 1 |
| *Strix nebulosa* | 3 | 712 | 349 | 695 | 349 | 0.2 | 324 | 0.655 | 2 | 2 |
| *Asio otus* | 3 | 685 | 338 | 684 | 339 | 2.106 | 870 | 0.147 | 1 | 3 |
| *Asio flammeus* | 2 | 709 | 343 | 703 | 343 | 19.062 | 958 | <0.001 | 3 | 3 |
| *Aegolius funereus* | 2 | 693 | 341 | 694 | 342 | 27.398 | 1224 | <0.001 | 2 | 2 |
| *Caprimulgus europaeus* | 2 | 678 | 337 | 676 | 339 | 26.658 | 576 | <0.001 | 2 | 4 |
| *Apus apus* | 2 | 696 | 343 | 696 | 343 | 13.861 | 1484 | <0.001 | 1 | 4 |
| *Alcedo atthis* | 3 | 670 | 338.5 | 681 | 337 | 2.325 | 42 | 0.135 | 3 | 3 |
| *Jynx torquilla* | 2 | 693 | 342 | 691 | 340 | 59.046 | 1336 | <0.001 | 2 | 4 |
| *Picus canus* | 1 | 674 | 333 | 679 | 339 | 118.4 | 590 | <0.001 | 2 | 1 |
| *Dryocopus martius* | 1 | 693 | 342 | 694 | 343 | 14.488 | 1380 | <0.001 | 2 | 1 |
| *Dendrocopos major* | 1 | 695.5 | 343 | 698 | 344 | 26.257 | 1546 | <0.001 | 2 | 1 |
| *Dendrocopos leucotos* | 1 | 682 | 344 | 682 | 345 | 85.324 | 266 | <0.001 | 2 | 1 |
| *Dendrocopos minor* | 1 | 686.5 | 340 | 685 | 338 | 31.603 | 970 | <0.001 | 2 | 1 |
| *Picoides tridactylus* | 1 | 700.5 | 343 | 698 | 343 | 18.057 | 1000 | <0.001 | 2 | 1 |
| *Lullula arborea* | 1 | 674 | 336 | 675 | 336 | 53.26 | 332 | <0.001 | 2 | 3 |
| *Alauda arvensis* | 2 | 694 | 342 | 690 | 340 | 79.133 | 1316 | <0.001 | 1 | 3 |
| *Riparia riparia* | 2 | 698 | 342 | 699 | 343 | 106.3 | 1276 | <0.001 | 3 | 4 |
| *Hirundo rustica* | 3 | 696 | 343 | 696 | 342.5 | 0.104 | 1494 | 0.747 | 1 | 4 |
| *Delichon urbicum* | 2 | 697 | 343 | 696 | 343 | 7.809 | 1550 | 0.005 | 1 | 4 |
| *Anthus trivialis* | 3 | 698 | 344 | 698 | 344 | 1.193 | 1590 | 0.275 | 2 | 4 |
| *Anthus prantensis* | 2 | 701 | 343 | 702 | 342 | 34.014 | 1588 | <0.001 | 3 | 3 |
| *Anthus cervinus* | 2 | 767 | 348 | 767 | 337 | 22.872 | 80 | <0.001 | 4 | 4 |
| *Anthus petrosus* | 3 | 670 | 320 | 671 | 320 | 1.048 | 120 | 0.308 | 5 | 3 |
| *Motacilla flava* | 2 | 701 | 344 | 712 | 345 | 130.62 | 1598 | <0.001 | 3 | 4 |
| *Motacilla cinerea* | 1 | 726 | 353 | 719 | 349.5 | 17.98 | 50 | <0.001 | 3 | 3 |
| *Motacilla citreola* | 1 | - | - | 670.5 | 337 | 5.311 | 14 | 0.037 | 3 | 4 |
| *Motacilla alba* | 3 | 700 | 344 | 699 | 344 | 1.084 | 1690 | 0.298 | 3 | 3 |
| *Bombycilla garrulus* | 1 | 735 | 354 | 729 | 348 | 140.96 | 738 | <0.001 | 2 | 3 |
| *Cinclus cinclus* | 1 | 757 | 348 | 751 | 351 | 12.427 | 154 | 0.001 | 3 | 2 |
| *Troglodytes troglodytes* | 1 | 687 | 343 | 689.5 | 343 | 51.89 | 1098 | <0.001 | 2 | 3 |
| *Prunella modularis* | 1 | 692 | 342 | 694 | 342 | 15.764 | 1394 | <0.001 | 2 | 3 |
| *Erithacus rubecula* | 1 | 694.5 | 342 | 696 | 343 | 9.489 | 1480 | 0.002 | 2 | 3 |
| *Luscinia luscinia* | 3 | 683 | 339 | 682.5 | 337 | 0.039 | 850 | 0.843 | 2 | 4 |
| *Luscinia svecica* | 2 | 753 | 348 | 759 | 349 | 58.821 | 392 | <0.001 | 4 | 4 |
| *Tarsiger cyanurus* | 1 | - | - | 719 | 358.5 | 35.662 | 88 | <0.001 | 2 | 4 |
| *Phoenicurus ochruros* | 1 | 680 | 330 | 682 | 337 | 44.899 | 110 | <0.001 | 1 | 3 |
| *Phoenicurus phoenicurus* | 3 | 700 | 344 | 700 | 344 | 0.849 | 1624 | 0.357 | 2 | 4 |
| *Saxicola rubetra* | 2 | 697 | 344 | 697 | 343 | 5.195 | 1522 | 0.023 | 1 | 4 |
| *Oenanthe oenanthe* | 2 | 699 | 343 | 697 | 341 | 119.04 | 1658 | <0.001 | 3 | 4 |
| *Turdus torquatus* | 3 | 767 | 347 | 767 | 337 | 0.274 | 16 | 0.608 | 4 | 3 |
| *Turdus merula* | 1 | 689 | 340 | 692 | 342 | 39.305 | 1260 | <0.001 | 2 | 2 |
| *Turdus pilaris* | 3 | 698 | 343 | 700 | 344 | 3.078 | 1638 | 0.08 | 1 | 3 |
| *Turdus philomelos* | 3 | 698.5 | 344 | 699 | 344 | 0.481 | 1624 | 0.488 | 2 | 3 |
| *Turdus iliacus* | 3 | 700 | 344 | 700 | 344 | 0.2 | 1648 | 0.655 | 2 | 3 |
| *Turdus viscivorus* | 1 | 696 | 344 | 699 | 345 | 75.954 | 1486 | <0.001 | 2 | 3 |
| *Locustella naevia* | 3 | 683 | 341 | 683 | 339 | 1.007 | 702 | 0.316 | 3 | 4 |
| *Locustella fluviatilis* | 1 | 678.5 | 339 | 682 | 342 | 17.087 | 472 | <0.001 | 3 | 4 |
| *Locustella luscinioides* | 1 | - | - | 677 | 342 | 17.087 | 38 | <0.001 | 3 | 4 |
| *Acrocephalus schoenobaenus* | 2 | 695 | 342 | 693 | 341 | 25.722 | 1400 | <0.001 | 3 | 4 |
| *Acrocephalus dumetorum* | 1 | 684 | 342 | 686 | 342 | 112.13 | 880 | <0.001 | 3 | 4 |
| *Acrocephalus palustris* | 1 | 678 | 338 | 680 | 337 | 25.471 | 650 | <0.001 | 3 | 4 |
| *Acrocephalus scirpaceus* | 3 | 676.5 | 335 | 677 | 334 | 0.015 | 418 | 0.903 | 3 | 4 |
| *Acrocephalus arundinaceus* | 1 | 675 | 341.5 | 676 | 337 | 4.898 | 210 | 0.028 | 3 | 4 |
| *Hippolais icterina* | 3 | 679 | 337 | 679 | 339 | 0.936 | 704 | 0.334 | 2 | 4 |
| *Sylvia borin* | 3 | 693 | 342 | 693 | 342 | 0.029 | 1344 | 0.864 | 2 | 4 |
| *Sylvia nisoria* | 2 | 668 | 333.5 | 668.5 | 329 | 18.075 | 78 | <0.001 | 5 | 4 |
| *Sylvia curruca* | 3 | 692 | 342 | 692 | 342 | 0.592 | 1340 | 0.442 | 2 | 4 |
| *Sylvia communis* | 3 | 689 | 340 | 689 | 340 | 0.127 | 1196 | 0.721 | 1 | 4 |
| *Sylvia atricapilla* | 1 | 681 | 337 | 684 | 329 | 37.995 | 920 | <0.001 | 2 | 4 |
| *Phylloscopus trochiloides* | 1 | 682 | 341.5 | 688 | 343.5 | 15.726 | 654 | <0.001 | 2 | 4 |
| *Phylloscopus borealis* | 2 | 713 | 353 | 730 | 359 | 18.583 | 148 | <0.001 | 2 | 4 |
| *Phylloscopus sibilatrix* | 2 | 690 | 342 | 690 | 341 | 6.115 | 1228 | 0.014 | 2 | 4 |
| *Phylloscopus collybita* | 2 | 693 | 342 | 692 | 341 | 5.247 | 1296 | 0.022 | 2 | 3 |
| *Phylloscopus trochilus* | 3 | 700 | 344 | 700 | 344 | 0.005 | 1682 | 0.941 | 2 | 4 |
| *Regulus regulus* | 1 | 693 | 343 | 695 | 343 | 20.464 | 1406 | <0.001 | 2 | 2 |
| *Muscicapa striata* | 3 | 698 | 344 | 698 | 344 | 0.149 | 1628 | 0.699 | 2 | 4 |
| *Ficedula parva* | 1 | 680 | 342 | 683 | 343 | 76.7 | 560 | <0.001 | 2 | 4 |
| *Ficedula hypoleuca* | 3 | 697 | 343 | 698 | 344 | 3.197 | 1596 | 0.074 | 2 | 4 |
| *Panurus biarmicus** | 1 | - | - | 671 | 324 | 33.029 | 35 | <0.001 | 3 | 2 |
| *Aegithalos caudatus* | 1 | 685 | 343 | 683 | 340 | 191.03 | 694 | <0.001 | 2 | 1 |
| *Poecile montanus* | 3 | 697 | 344 | 697 | 343 | 0.645 | 1520 | 0.422 | 2 | 1 |
| *Poecile cinctus* | 3 | 751 | 350 | 754 | 350 | 0.074 | 352 | 0.786 | 2 | 1 |
| *Lophophanes cristatus* | 1 | 690 | 341 | 690 | 342 | 4.624 | 1178 | 0.032 | 2 | 1 |
| *Periparus ater* | 1 | 681 | 335 | 685 | 338 | 76.909 | 902 | <0.001 | 2 | 1 |
| *Cyanistes caeruleus* | 1 | 684 | 337 | 693 | 342 | 149.1 | 902 | <0.001 | 2 | 2 |
| *Parus major* | 1 | 696 | 342 | 698 | 344 | 22.69 | 1574 | <0.001 | 2 | 2 |
| *Sitta europaea* | 3 | 681.5 | 340 | 693 | 351 | 0.08 | 48 | 0.778 | 2 | 1 |
| *Certhia familiaris* | 1 | 689 | 340 | 691 | 342 | 36.534 | 1206 | <0.001 | 2 | 2 |
| *Oriolus oriolus* | 2 | 682 | 346 | 681 | 347 | 60.718 | 490 | <0.001 | 2 | 4 |
| *Lanius collurio* | 2 | 688 | 340 | 687.5 | 339.5 | 8.597 | 1140 | 0.003 | 2 | 4 |
| *Lanius excubitor* | 3 | 706 | 344 | 703 | 342 | 3.567 | 976 | 0.059 | 2 | 3 |
| *Garrulus glandarius* | 3 | 692 | 342 | 693 | 342 | 25.343 | 246 | <0.001 | 2 | 1 |
| *Perisoreus infaustus* | 3 | 734.5 | 348.5 | 739 | 351 | 2.405 | 668 | 0.121 | 2 | 1 |
| *Pica pica* | 3 | 696 | 342.5 | 696 | 343 | 0.007 | 1440 | 0.934 | 1 | 1 |
| *Nucifraga caryocatactes* | 1 | 676 | 332 | 683.5 | 332 | 25.343 | 246 | <0.001 | 2 | 1 |
| *Corvus monedula* | 1 | 682 | 335.5 | 689 | 338 | 76.376 | 910 | <0.001 | 1 | 2 |
| *Corvus frugilegus* | 3 | 684 | 335 | 696.5 | 337 | 0.035 | 116 | 0.852 | 1 | 3 |
| *Corvus corone* | 3 | 698 | 343 | 697 | 343 | 0.986 | 1652 | 0.321 | 1 | 2 |
| *Corvus corax* | 1 | 699 | 343.5 | 699 | 344 | 11.414 | 1630 | 0.001 | 2 | 1 |
| *Sturnus vulgaris* | 2 | 692 | 341 | 687 | 338 | 99.179 | 1280 | <0.001 | 1 | 3 |
| *Passer domesticus* | 2 | 692 | 341 | 692 | 341 | 12.029 | 1248 | 0.001 | 1 | 1 |
| *Passer montanus* | 1 | 678.5 | 350 | 684 | 337.5 | 245.49 | 734 | <0.001 | 1 | 1 |
| *Fringilla coelebs* | 3 | 697 | 343 | 697 | 343 | 0.154 | 1545 | 0.695 | 2 | 3 |
| *Fringilla montifringilla* | 2 | 704 | 345 | 721 | 346 | 169.16 | 1488 | <0.001 | 2 | 3 |
| *Serinus serinus* | 3 | 693.5 | 337 | 674 | 339 | 0.167 | 22 | 0.687 | 1 | 3 |
| *Carduelis chloris* | 1 | 689 | 339 | 695 | 342 | 108.78 | 1400 | <0.001 | 1 | 2 |
| *Carduelis carduelis* | 1 | 676 | 338 | 677 | 337 | 68.35 | 408 | <0.001 | 1 | 3 |
| *Carduelis spinus* | 1 | 696 | 343 | 698 | 344 | 28.147 | 1580 | <0.001 | 2 | 3 |
| *Carduelis cannabina* | 2 | 681 | 336 | 677 | 335 | 47.518 | 672 | <0.001 | 1 | 3 |
| *Carduelis flammea* | 2 | 711.5 | 347 | 725 | 345 | 98.741 | 1326 | <0.001 | 2 | 2 |
| *Loxia leucoptera* | 2 | 734 | 347.5 | 741 | 354 | 12.493 | 216 | <0.001 | 2 | 2 |
| *Loxia curvirostra* | 3 | 696 | 343 | 697 | 343 | 1.098 | 1510 | 0.295 | 2 | 2 |
| *Loxia pytyopsittacus* | 3 | 694 | 343 | 702 | 345 | 0.647 | 1024 | 0.421 | 2 | 2 |
| *Carpodacus erythrinus* | 2 | 693 | 342 | 693 | 342 | 6.883 | 1304 | 0.009 | 2 | 4 |
| *Pinicola enucleator* | 2 | 753 | 351 | 754.5 | 353 | 14.653 | 228 | <0.001 | 2 | 2 |
| *Pyrrhula pyrrhula* | 3 | 696 | 343 | 696 | 344 | 1.287 | 1456 | 0.257 | 2 | 2 |
| *Coccothraustes coccothraustes* | 1 | 677 | 338 | 678 | 337 | 53.333 | 222 | <0.001 | 2 | 3 |
| *Calcarius lapponicus* | 2 | 763 | 346 | 767 | 345 | 14.185 | 140 | <0.001 | 4 | 3 |
| *Plectrophenax nivalis* | 2 | 765.5 | 347 | 767 | 346 | 5.454 | 68 | 0.022 | 4 | 3 |
| *Emberiza citrinella* | 3 | 695 | 343 | 695 | 342.5 | 0 | 1424 | 1 | 1 | 2 |
| *Emberiza hortulana* | 2 | 692 | 341 | 682 | 336 | 212.13 | 1096 | <0.001 | 1 | 4 |
| *Emberiza rustica* | 2 | 702.5 | 346 | 714 | 348 | 218.69 | 1156 | <0.001 | 2 | 4 |
| *Emberiza pusilla* | 2 | 747 | 352 | 743 | 349 | 4.045 | 150 | 0.046 | 2 | 4 |
| *Emberiza aureola** | 2 | 697 | 356 | - | - | 33.029 | 35 | <0.001 | 3 | 4 |
| *Emberiza schoeniclus* | 3 | 700 | 344 | 699.5 | 344 | 2.888 | 1622 | 0.089 | 3 | 3 |
