# Supplementary material for: Birds on the move in the face of climate change: High species turnover in northern Europe
Source: Ecol Evol. 2017 Sep 6;7(20):8201–9. doi: 10.1002/ece3.3328 (PMC5648647; doi:10.1002/ece3.3328)
Supplement: Supplementary file 2 [file ECE3-7-8201-s002.docx]

Table S2. Comparison of the model explaining species-specific variation in the range size change using different phylogenic trees. The phylogenies of the trees are given in the footnote of the table.

| Tree | DIC | ∆DIC |
| --- | --- | --- |
| TREE9 | 4288.412 | 0 |
| TREE1 | 4288.896 | 0.484 |
| TREE2 | 4289.402 | 0.99 |
| TREE5 | 4289.518 | 1.106 |
| TREE4 | 4289.574 | 1.162 |
| TREE8 | 4289.585 | 1.173 |
| TREE3 | 4289.724 | 1.312 |
| TREE10 | 4289.788 | 1.376 |
| TREE7 | 4289.868 | 1.456 |
| TREE6 | 4290.188 | 1.776 |

TREE1=(((((Cygnus_olor:4.724015,Cygnus_cygnus:4.724015):3.105033,((Anser_fabalis:1.395563,Anser_anser:1.395563):2.910609,(Branta_leucopsis:1.235615,Branta_canadensis:1.235615):3.070556):3.522878):5.507380,(((((Anas_crecca:2.323762,Anas_acuta:2.323762):0.140439,Anas_platyrhynchos:2.464201):0.579979,(Anas_strepera:1.597176,Anas_penelope:1.597176):1.447005):1.162668,(Anas_querquedula:2.106736,Anas_clypeata:2.106736):2.100113):2.357848,((((Clangula_hyemalis:5.127074,Somateria_mollissima:5.127074):0.809644,((Melanitta_nigra:3.422698,Melanitta_fusca:3.422698):1.560144,((Mergellus_albellus:4.207836,(Mergus_serrator:0.674720,Mergus_merganser:0.674720):3.533116):0.391998,Bucephala_clangula:4.599834):0.383009):0.953875):0.179889,Tadorna_tadorna:6.116606):0.132588,((Aythya_marila:0.687473,Aythya_fuligula:0.687473):0.215079,Aythya_ferina:0.902552):5.346642):0.315503):6.771732):62.989662,((Bonasa_bonasia:13.125723,((Tetrao_tetrix:4.194616,Tetrao_urogallus:4.194616):3.548759,(Lagopus_muta:3.893541,Lagopus_lagopus:3.893541):3.849834):5.382347):4.625540,(Phasianus_colchicus:15.895084,Perdix_perdix:15.895083):1.856179):58.574837):20.442041,(((Streptopelia_decaocto:9.255561,Streptopelia_turtur:9.255561):10.197813,((Columba_livia:8.666072,Columba_oenas:8.666072):2.884518,Columba_palumbus:11.550590):7.902785):66.335182,((((Ardea_cinerea:43.432278,Botaurus_stellaris:43.432278):28.083050,((Gavia_stellata:48.539421,Gavia_arctica:48.539421):19.631300,Phalacrocorax_carbo:68.170723):3.344604):2.657127,((((((Eudromias_morinellus:32.551567,(Charadrius_dubius:27.261398,Charadrius_hiaticula:27.261398):5.290168):6.032756,Vanellus_vanellus:38.584328):11.235333,Haematopus_ostralegus:49.819656):1.066525,Pluvialis_apricaria:50.886181):12.973968,(((Numenius_phaeopus:12.786203,Numenius_arquata:12.786203):16.727242,(((Phalaropus_lobatus:21.175905,((((Tringa_erythropus:8.983128,Tringa_nebularia:8.983128):2.036585,((Tringa_totanus:5.484750,Tringa_glareola:5.484750):0.659190,Tringa_stagnatilis:6.143939):4.875772):3.405485,Tringa_ochropus:14.425197):5.553394,Actitis_hypoleucos:19.978592):1.197315):4.461888,((((Calidris_alpina:12.660132,Calidris_temminckii:12.660132):1.824120,(Limicola_falcinellus:14.255639,Philomachus_pugnax:14.255639):0.228613):7.413331,Arenaria_interpres:21.897585):3.413976,(Scolopax_rusticola:19.652676,(Gallinago_gallinago:10.645719,Gallinago_media:10.645718):9.006957):5.658885):0.326234):1.584344,((Limosa_lapponica:11.487151,Limosa_limosa:11.487152):12.202610,Lymnocryptes_minimus:23.689760):3.532377):2.291308):18.780703,(((((((Larus_argentatus:0.677651,Larus_marinus:0.677651):0.140639,Larus_fuscus:0.818290):0.452072,Larus_canus:1.270362):3.260170,Larus_ridibundus:4.530532):0.379169,Larus_minutus:4.909701):11.548464,(Sterna_albifrons:10.994645,(Sterna_caspia:7.572935,(Chlidonias_niger:6.985326,(Sterna_paradisaea:2.327330,Sterna_hirundo:2.327330):4.657996):0.587608):3.421710):5.463520):2.931790,((Stercorarius_longicaudus:2.001547,Stercorarius_parasiticus:2.001547):15.204206,(Alca_torda:13.344406,Cepphus_grylle:13.344406):3.861348):2.184201):28.904194):15.566001):5.918958,((((Podiceps_cristatus:6.423506,Podiceps_auritus:6.423506):2.469896,Podiceps_grisegena:8.893402):15.121864,Tachybaptus_ruficollis:24.015266):40.676193,(((Rallus_aquaticus:16.635506,Crex_crex:16.635508):9.962881,(Porzana_parva:25.061352,(Porzana_porzana:17.162754,(Fulica_atra:11.557182,Gallinula_chloropus:11.557182):5.605571):7.898598):1.537036):19.815943,Grus_grus:46.414333):18.277128):5.087647):4.393346):10.099190,(Cuculus_canorus:84.206238,(((Falco_tinnunculus:15.800543,(Falco_columbarius:13.425261,((Falco_rusticolus:4.607901,Falco_peregrinus:4.607901):7.125186,Falco_subbuteo:11.733087):1.692175):2.375281):63.770943,((Oriolus_oriolus:35.013412,(((((Nucifraga_caryocatactes:14.995872,(((Corvus_frugilegus:6.939942,Corvus_corax:6.939941):0.690668,Corvus_corone:7.630609):3.889558,Corvus_monedula:11.520167):3.475706):1.254080,Pica_pica:16.249954):1.865057,Garrulus_glandarius:18.115009):3.503951,Perisoreus_infaustus:21.618961):7.991398,(Lanius_collurio:12.515026,Lanius_excubitor:12.515026):17.095333):5.403053):17.355949,((((Parus_major:15.719477,(((Parus_cinctus:10.024444,Parus_montanus:10.024444):4.907726,Parus_ater:14.932170):0.117473,Parus_cristatus:15.049643):0.669835):2.110568,Parus_caeruleus:17.830046):29.645378,((Panurus_biarmicus:33.220520,(Alauda_arvensis:12.307558,Lullula_arborea:12.307558):20.912964):9.772917,(((((Hirundo_rustica:19.346647,Delichon_urbicum:19.346649):4.261378,Riparia_riparia:23.608027):13.313770,((Hippolais_icterina:18.167091,(Acrocephalus_arundinaceus:15.729233,((Acrocephalus_scirpaceus:8.720958,Acrocephalus_dumetorum:8.720958):4.335438,Acrocephalus_schoenobaenus:13.056396):2.672838):2.437856):16.927382,((Locustella_naevia:12.096982,(Locustella_luscinioides:7.257441,Locustella_fluviatilis:7.257441):4.839541):11.753009,Acrocephalus_palustris:23.849991):11.244481):1.827324):0.061485,(((Phylloscopus_trochiloides:12.612716,Phylloscopus_borealis:12.612716):7.280677,((Phylloscopus_trochilus:6.046161,Phylloscopus_collybita:6.046161):10.265133,Phylloscopus_sibilatrix:16.311295):3.582099):7.672139,Aegithalos_caudatus:27.565533):9.417749):2.118387,((Sylvia_borin:15.056934,Sylvia_atricapilla:15.056933):2.955318,((Sylvia_nisoria:10.452719,Sylvia_curruca:10.452719):0.316624,Sylvia_communis:10.769342):7.242909):21.089417):3.891770):4.481989):1.185499,((Regulus_regulus:45.935375,((((Certhia_familiaris:33.545906,Troglodytes_troglodytes:33.545906):6.274453,Sitta_europaea:39.820358):3.889085,(Sturnus_vulgaris:30.231590,(Cinclus_cinclus:29.312300,((Turdus_viscivorus:16.903128,((((Turdus_pilaris:6.958612,Turdus_torquatus:6.958612):5.706686,Turdus_iliacus:12.665298):0.260532,Turdus_merula:12.925831):3.636420,Turdus_philomelos:16.562250):0.340878):11.024218,((Muscicapa_striata:20.831593,(Erithacus_rubecula:19.324381,(((Ficedula_parva:9.405882,Ficedula_hypoleuca:9.405883):8.371422,((Phoenicurus_ochruros:7.238223,Phoenicurus_phoenicurus:7.238223):8.955692,(Saxicola_rubetra:14.325880,Oenanthe_oenanthe:14.325880):1.868036):1.583387):1.045071,(Luscinia_luscinia:14.027993,Luscinia_svecica:14.027992):4.794382):0.502007):1.507209):1.648990,Tarsiger_cyanurus:22.480581):5.446765):1.384953):0.919291):13.477854):1.144202,Bombycilla_garrulus:44.853645):1.081730):0.864728,(Prunella_modularis:36.232426,((Passer_montanus:5.658614,Passer_domesticus:5.658614):27.899364,(((Anthus_trivialis:8.309520,((Anthus_petrosus:2.522432,Anthus_pratensis:2.522432):1.576368,Anthus_cervinus:4.098800):4.210720):14.069154,(Motacilla_cinerea:4.631705,((Motacilla_citreola:4.016164,Motacilla_flava:4.016164):0.267344,Motacilla_alba:4.283507):0.348198):17.746969):9.376805,(((((Carpodacus_erythrinus:17.790630,(Carduelis_chloris:11.527118,((Carduelis_carduelis:10.183993,(Carduelis_flammea:6.849329,((Loxia_pytyopsittacus:0.573335,Loxia_curvirostra:0.573335):1.605668,Loxia_leucoptera:2.179003):4.670326):3.334665):0.170989,(Serinus_serinus:10.244068,(Carduelis_cannabina:8.072887,Carduelis_spinus:8.072887):2.171181):0.110915):1.172135):6.263513):0.376432,(Pyrrhula_pyrrhula:14.883782,Pinicola_enucleator:14.883782):3.283281):4.624612,Coccothraustes_coccothraustes:22.791676):3.893307,(Fringilla_coelebs:7.027924,Fringilla_montifringilla:7.027924):19.657059):2.028887,(((Emberiza_hortulana:10.236762,Emberiza_citrinella:10.236762):8.554516,(((Emberiza_aureola:9.238657,Emberiza_pusilla:9.238657):1.381803,Emberiza_rustica:10.620461):1.686011,Emberiza_schoeniclus:12.306472):6.484806):5.955622,(Calcarius_lapponicus:22.342472,Plectrophenax_nivalis:22.342474):2.404427):3.966969):3.041610):1.802500):2.674445):10.567678):1.860823):3.708435):27.202127):2.661976,(Apus_apus:82.145721,(((Pernis_apivorus:42.106991,((((Milvus_migrans:18.506950,Haliaeetus_albicilla:18.506950):3.729777,(Buteo_lagopus:2.990799,Buteo_buteo:2.990799):19.245930):5.950038,((Accipiter_gentilis:18.646229,((Circus_pygargus:3.581905,Circus_cyaneus:3.581905):4.881339,Circus_aeruginosus:8.463244):10.182983):3.020539,Accipiter_nisus:21.666765):6.520000):4.275421,Aquila_chrysaetos:32.462189):9.644803):19.972933,Pandion_haliaetus:62.079926):16.860052,(((((((Strix_uralensis:9.976760,Strix_aluco:9.976760):4.212125,Strix_nebulosa:14.188885):11.359199,(Bubo_scandiaca:13.083157,Bubo_bubo:13.083157):12.464928):6.760704,(Asio_otus:14.537540,Asio_flammeus:14.537542):17.771248):10.088024,((Surnia_ulula:22.236012,Glaucidium_passerinum:22.236012):9.734512,Aegolius_funereus:31.970524):10.426288):34.252304,((((Picus_canus:8.864795,Dryocopus_martius:8.864795):5.050727,(Picoides_tridactylus:11.973474,(Dendrocopos_minor:9.120564,(Dendrocopos_leucotos:2.352621,Dendrocopos_major:2.352621):6.767943):2.852909):1.942048):9.611070,Jynx_torquilla:23.526590):37.984955,Alcedo_atthis:61.511539):15.137571):1.352007,Caprimulgus_europaeus:78.001129):0.938854):3.205747):0.087742):1.972773):0.065404):1.516912):10.979580):6.425853;

TREE2=(((((Cygnus_cygnus:8.362403,Cygnus_olor:8.362403):5.700988,((Anser_fabalis:1.372808,Anser_anser:1.372808):7.490295,(Branta_leucopsis:2.077316,Branta_canadensis:2.077316):6.785787):5.200288):13.452543,((((((Melanitta_nigra:6.667158,Melanitta_fusca:6.667158):2.352586,((Mergellus_albellus:5.681093,(Mergus_merganser:1.695604,Mergus_serrator:1.695604):3.985489):2.884903,Bucephala_clangula:8.565996):0.453748):1.747990,(Clangula_hyemalis:10.118098,Somateria_mollissima:10.118098):0.649635):0.625802,Tadorna_tadorna:11.393536):0.090246,((Aythya_marila:1.378258,Aythya_fuligula:1.378259):0.949249,Aythya_ferina:2.327507):9.156275):1.322508,(((Anas_acuta:4.811951,(Anas_platyrhynchos:4.664622,Anas_crecca:4.664622):0.147328):1.908812,(Anas_penelope:3.138559,Anas_strepera:3.138559):3.582204):1.895938,(Anas_querquedula:3.645814,Anas_clypeata:3.645813):4.970887):4.189589):14.709644):58.735050,((((Lagopus_muta:3.706849,Lagopus_lagopus:3.706849):5.626869,(Tetrao_tetrix:4.990723,Tetrao_urogallus:4.990723):4.342996):3.068711,Bonasa_bonasia:12.402429):6.847040,(Phasianus_colchicus:16.223684,Perdix_perdix:16.223686):3.025783):67.001511):16.166725,(((Streptopelia_decaocto:8.699877,Streptopelia_turtur:8.699877):7.994635,(Columba_palumbus:9.390046,(Columba_livia:6.288736,Columba_oenas:6.288736):3.101310):7.304465):71.753342,((((Ardea_cinerea:34.699760,Botaurus_stellaris:34.699760):31.029770,((Gavia_stellata:44.639172,Gavia_arctica:44.639172):19.778744,Phalacrocorax_carbo:64.417923):1.311619):12.391313,(((Tachybaptus_ruficollis:34.696991,((Podiceps_cristatus:11.238394,Podiceps_grisegena:11.238394):2.150745,Podiceps_auritus:13.389138):21.307850):38.823097,(((Crex_crex:21.044106,Rallus_aquaticus:21.044106):15.176890,((Porzana_porzana:19.587246,(Fulica_atra:14.071425,Gallinula_chloropus:14.071425):5.515821):10.933884,Porzana_parva:30.521128):5.699867):13.452127,Grus_grus:49.673122):23.846964):3.252733,(((((Eudromias_morinellus:33.504074,(Charadrius_hiaticula:26.601734,Charadrius_dubius:26.601736):6.902339):5.038862,Vanellus_vanellus:38.542938):3.687519,Haematopus_ostralegus:42.230453):2.461438,Pluvialis_apricaria:44.691895):20.934589,(((Numenius_phaeopus:16.227657,Numenius_arquata:16.227657):28.980156,((Lymnocryptes_minimus:28.410927,(Limosa_lapponica:13.128526,Limosa_limosa:13.128526):15.282402):9.556709,((Arenaria_interpres:27.565670,((Calidris_alpina:16.222357,Calidris_temminckii:16.222357):3.041023,(Philomachus_pugnax:16.571398,Limicola_falcinellus:16.571398):2.691982):8.302289):8.314598,((Scolopax_rusticola:29.959681,(Gallinago_media:13.178608,Gallinago_gallinago:13.178609):16.781073):4.756493,(Phalaropus_lobatus:30.446772,(Actitis_hypoleucos:29.687969,(Tringa_ochropus:22.443130,((Tringa_totanus:8.900353,(Tringa_glareola:8.857835,Tringa_stagnatilis:8.857835):0.042519):6.037864,(Tringa_nebularia:12.212120,Tringa_erythropus:12.212119):2.726098):7.504914):7.244838):0.758802):4.269403):1.164093):2.087370):7.240175):11.943682,(((Sterna_albifrons:11.653790,(Sterna_caspia:8.716401,(Chlidonias_niger:7.421080,(Sterna_paradisaea:3.202021,Sterna_hirundo:3.202021):4.219059):1.295322):2.937389):5.847329,(Larus_minutus:7.019364,(Larus_ridibundus:4.193777,(Larus_canus:1.718462,(Larus_fuscus:0.939478,(Larus_argentatus:0.761640,Larus_marinus:0.761640):0.177838):0.778984):2.475315):2.825588):10.481754):3.061999,((Alca_torda:14.298271,Cepphus_grylle:14.298271):4.781960,(Stercorarius_parasiticus:3.150835,Stercorarius_longicaudus:3.150835):15.929398):1.482887):36.588375):8.474991):11.146336):1.348027):9.474976,(Cuculus_canorus:87.363853,((((Oriolus_oriolus:31.971933,((Lanius_collurio:9.421520,Lanius_excubitor:9.421521):18.125406,(((Pica_pica:14.807585,(Nucifraga_caryocatactes:13.053061,(Corvus_monedula:9.041737,((Corvus_frugilegus:4.860324,Corvus_corax:4.860324):1.860081,Corvus_corone:6.720406):2.321331):4.011323):1.754525):1.151413,Garrulus_glandarius:15.958998):3.986826,Perisoreus_infaustus:19.945824):7.601103):4.425006):16.960726,(((Parus_caeruleus:16.838078,(((Parus_ater:14.283553,Parus_major:14.283552):0.522352,(Parus_montanus:10.254998,Parus_cinctus:10.254999):4.550906):0.572961,Parus_cristatus:15.378866):1.459212):21.389389,((Panurus_biarmicus:26.779455,(Lullula_arborea:8.955973,Alauda_arvensis:8.955972):17.823483):7.400646,(((((((Phylloscopus_collybita:6.190758,Phylloscopus_trochilus:6.190757):8.641603,Phylloscopus_sibilatrix:14.832361):1.927739,(Phylloscopus_trochiloides:10.367975,Phylloscopus_borealis:10.367975):6.392125):7.386852,Aegithalos_caudatus:24.146954):5.053215,((((Locustella_luscinioides:5.030581,Locustella_fluviatilis:5.030581):4.103095,Locustella_naevia:9.133675):8.726179,Acrocephalus_palustris:17.859854):9.379948,((Acrocephalus_arundinaceus:10.346318,((Acrocephalus_dumetorum:5.751252,Acrocephalus_scirpaceus:5.751252):3.577600,Acrocephalus_schoenobaenus:9.328852):1.017467):2.004230,Hippolais_icterina:12.350548):14.889254):1.960366):1.054124,((Hirundo_rustica:18.428253,Delichon_urbicum:18.428251):2.493775,Riparia_riparia:20.922028):9.332265):1.826410,((Sylvia_nisoria:10.892706,(Sylvia_communis:10.406010,Sylvia_curruca:10.406010):0.486696):2.561308,(Sylvia_atricapilla:9.608212,Sylvia_borin:9.608212):3.845803):18.626686):2.099399):4.047366):4.405388,(((((Sturnus_vulgaris:25.172928,(Cinclus_cinclus:23.326145,(((Turdus_philomelos:11.785146,Turdus_viscivorus:11.785146):0.764461,((Turdus_pilaris:5.653997,Turdus_torquatus:5.653997):4.169829,(Turdus_iliacus:9.818912,Turdus_merula:9.818912):0.004915):2.725781):8.018096,(Tarsiger_cyanurus:15.068437,(Muscicapa_striata:13.955524,((((Ficedula_hypoleuca:6.729757,Ficedula_parva:6.729757):5.106253,((Phoenicurus_ochruros:4.816994,Phoenicurus_phoenicurus:4.816994):5.495733,(Oenanthe_oenanthe:9.425464,Saxicola_rubetra:9.425465):0.887263):1.523283):0.820554,(Luscinia_svecica:10.719051,Luscinia_luscinia:10.719051):1.937514):0.132441,Erithacus_rubecula:12.789005):1.166520):1.112912):5.499266):2.758440):1.846784):11.722316,((Certhia_familiaris:23.879116,Troglodytes_troglodytes:23.879116):2.894399,Sitta_europaea:26.773516):10.121729):1.342486,Bombycilla_garrulus:38.237728):2.746969,Regulus_regulus:40.984699):1.381168,(((Passer_montanus:7.926940,Passer_domesticus:7.926940):20.175259,((((Coccothraustes_coccothraustes:18.560905,(((Pyrrhula_pyrrhula:11.392794,Pinicola_enucleator:11.392793):3.799281,Carpodacus_erythrinus:15.192074):0.456795,(((Carduelis_cannabina:6.916501,Carduelis_spinus:6.916501):0.837340,(((Carduelis_flammea:4.963035,((Loxia_curvirostra:0.391200,Loxia_pytyopsittacus:0.391200):0.699389,Loxia_leucoptera:1.090589):3.872446):2.042189,Carduelis_carduelis:7.005224):0.743074,Serinus_serinus:7.748298):0.005543):2.743295,Carduelis_chloris:10.497136):5.151733):2.912038):4.352264,(Fringilla_coelebs:7.668959,Fringilla_montifringilla:7.668960):15.244211):1.250589,((Calcarius_lapponicus:14.162357,Plectrophenax_nivalis:14.162356):5.318798,((Emberiza_hortulana:7.091859,Emberiza_citrinella:7.091858):4.841215,(Emberiza_schoeniclus:8.191181,(Emberiza_rustica:7.225412,(Emberiza_pusilla:5.344007,Emberiza_aureola:5.344007):1.881405):0.965769):3.741892):7.548080):4.682605):3.254070,(((Motacilla_alba:3.150697,(Motacilla_citreola:1.841688,Motacilla_flava:1.841688):1.309008):0.330996,Motacilla_cinerea:3.481693):15.342262,(Anthus_trivialis:9.365601,((Anthus_petrosus:2.244725,Anthus_pratensis:2.244725):2.089603,Anthus_cervinus:4.334329):5.031271):9.458356):8.593874):0.684370):5.635007,Prunella_modularis:33.737206):8.628659):0.266988):6.299806):34.145100,((Falco_columbarius:12.901619,Falco_tinnunculus:12.901620):0.705905,(Falco_subbuteo:11.281702,(Falco_rusticolus:3.630489,Falco_peregrinus:3.630489):7.651213):2.325822):69.470238):3.069522,(Apus_apus:85.537064,((((((Asio_flammeus:16.499165,Asio_otus:16.499163):19.194464,((Strix_nebulosa:14.920827,(Strix_aluco:11.826944,Strix_uralensis:11.826944):3.093884):10.191261,(Bubo_scandiaca:14.800200,Bubo_bubo:14.800200):10.311889):10.581538):10.141438,((Surnia_ulula:27.435751,Glaucidium_passerinum:27.435753):7.635045,Aegolius_funereus:35.070797):10.764267):34.669472,((((Picus_canus:8.648508,Dryocopus_martius:8.648508):2.777502,(Picoides_tridactylus:9.071449,((Dendrocopos_leucotos:2.233722,Dendrocopos_major:2.233722):5.040161,Dendrocopos_minor:7.273883):1.797567):2.354560):8.579658,Jynx_torquilla:20.005669):45.851402,Alcedo_atthis:65.857079):14.647461):1.136494,Caprimulgus_europaeus:81.641029):0.018484,((Pernis_apivorus:45.698460,(Aquila_chrysaetos:37.618374,((Accipiter_nisus:27.066578,(Accipiter_gentilis:23.324158,(Circus_cyaneus:11.752390,(Circus_aeruginosus:7.330950,Circus_pygargus:7.330950):4.421440):11.571768):3.742420):6.711445,((Milvus_migrans:21.109694,Haliaeetus_albicilla:21.109692):4.801130,(Buteo_lagopus:2.859386,Buteo_buteo:2.859385):23.051437):7.867198):3.840353):8.080082):18.930750,Pandion_haliaetus:64.629204):17.030306):3.877545):0.610224):1.216574):0.231967):0.852026):13.969858):0.172585;

TREE3=(((((Cygnus_cygnus:7.237604,Cygnus_olor:7.237604):2.091574,((Anser_anser:0.980908,Anser_fabalis:0.980908):6.104393,(Branta_canadensis:1.017525,Branta_leucopsis:1.017525):6.067776):2.243877):7.869722,(((((Anas_crecca:2.745383,Anas_platyrhynchos:2.745383):0.069250,Anas_acuta:2.814633):0.901176,(Anas_penelope:1.901188,Anas_strepera:1.901188):1.814621):1.343006,(Anas_clypeata:2.516810,Anas_querquedula:2.516810):2.542006):3.375939,(((Aythya_marila:1.001226,Aythya_fuligula:1.001226):0.474808,Aythya_ferina:1.476034):6.643785,(((Clangula_hyemalis:6.575243,Somateria_mollissima:6.575243):0.394358,((Melanitta_nigra:5.049791,Melanitta_fusca:5.049791):0.732015,((Mergellus_albellus:4.668801,(Mergus_merganser:1.027941,Mergus_serrator:1.027941):3.640861):0.511659,Bucephala_clangula:5.180461):0.601345):1.187795):0.541603,Tadorna_tadorna:7.511204):0.608615):0.314936):8.764146):55.331894,((((Tetrao_urogallus:2.777699,Tetrao_tetrix:2.777699):2.825351,(Lagopus_muta:1.728278,Lagopus_lagopus:1.728278):3.874774):3.132725,Bonasa_bonasia:8.735777):3.806271,(Phasianus_colchicus:10.967090,Perdix_perdix:10.967090):1.574957):59.988747):28.251623,(((Streptopelia_decaocto:11.515139,Streptopelia_turtur:11.515139):7.704109,((Columba_oenas:6.957862,Columba_livia:6.957863):4.155093,Columba_palumbus:11.112957):8.106293):66.657089,(((((Gavia_stellata:46.651058,Gavia_arctica:46.651058):25.851608,Phalacrocorax_carbo:72.502663):0.982219,(Botaurus_stellaris:47.948574,Ardea_cinerea:47.948574):25.536308):6.938303,(((((Vanellus_vanellus:40.519119,((Charadrius_dubius:30.617844,Charadrius_hiaticula:30.617844):6.159790,Eudromias_morinellus:36.777634):3.741481):9.703224,Haematopus_ostralegus:50.222340):1.109546,Pluvialis_apricaria:51.331886):19.349798,(((Numenius_phaeopus:17.217289,Numenius_arquata:17.217289):24.629036,((Lymnocryptes_minimus:30.545883,(Limosa_lapponica:14.728951,Limosa_limosa:14.728952):15.816931):7.579075,((Arenaria_interpres:31.856251,((Calidris_alpina:17.161041,Calidris_temminckii:17.161039):2.241998,(Philomachus_pugnax:17.859850,Limicola_falcinellus:17.859850):1.543190):12.453213):4.698649,((Actitis_hypoleucos:32.444931,((Tringa_ochropus:21.686314,((Tringa_stagnatilis:8.333031,(Tringa_glareola:8.015559,Tringa_totanus:8.015559):0.317472):7.324830,(Tringa_erythropus:11.673149,Tringa_nebularia:11.673149):3.984712):6.028453):9.423853,Phalaropus_lobatus:31.110167):1.334764):3.558834,(Scolopax_rusticola:28.960266,(Gallinago_gallinago:15.584668,Gallinago_media:15.584668):13.375597):7.043499):0.551136):1.570058):3.721366):24.821226,(((Larus_minutus:5.293251,(Larus_ridibundus:4.379439,(Larus_canus:1.485547,(Larus_marinus:0.903134,(Larus_argentatus:0.890372,Larus_fuscus:0.890372):0.012762):0.582414):2.893892):0.913812):12.327785,(Sterna_albifrons:11.357853,(((Sterna_paradisaea:4.084692,Sterna_hirundo:4.084693):3.100775,Chlidonias_niger:7.185467):1.416457,Sterna_caspia:8.601924):2.755929):6.263183):2.511575,((Stercorarius_longicaudus:4.462640,Stercorarius_parasiticus:4.462641):14.075130,(Cepphus_grylle:13.369795,Alca_torda:13.369794):5.167977):1.594840):46.534943):4.014132):6.483714,(((((Gallinula_chloropus:13.397879,Fulica_atra:13.397879):9.405798,(Porzana_parva:17.629078,Porzana_porzana:17.629078):5.174599):11.669080,(Rallus_aquaticus:17.567570,Crex_crex:17.567570):16.905186):17.995737,Grus_grus:52.468491):23.618849,((Podiceps_cristatus:14.715230,(Podiceps_grisegena:10.551796,Podiceps_auritus:10.551796):4.163435):25.316013,Tachybaptus_ruficollis:40.031242):36.056099):1.078054):3.257789):1.585826,(Cuculus_canorus:81.358948,((((((Lanius_collurio:10.288605,Lanius_excubitor:10.288605):15.243297,((Garrulus_glandarius:16.810566,((Nucifraga_caryocatactes:12.637491,(((Corvus_frugilegus:4.576263,Corvus_corax:4.576262):1.466156,Corvus_corone:6.042418):3.695936,Corvus_monedula:9.738355):2.899137):1.845062,Pica_pica:14.482553):2.328014):1.501868,Perisoreus_infaustus:18.312435):7.219467):2.313582,Oriolus_oriolus:27.845482):14.878717,((((((Parus_cristatus:14.111158,(Parus_cinctus:10.361839,Parus_montanus:10.361839):3.749320):0.265639,Parus_ater:14.376797):0.965352,Parus_major:15.342150):1.467755,Parus_caeruleus:16.809906):20.221714,((Panurus_biarmicus:28.689787,(Lullula_arborea:9.525930,Alauda_arvensis:9.525929):19.163857):4.380743,((((Sylvia_curruca:8.783653,Sylvia_nisoria:8.783653):1.353283,Sylvia_communis:10.136937):4.655138,(Sylvia_borin:11.838619,Sylvia_atricapilla:11.838619):2.953456):13.531595,(((((Phylloscopus_trochiloides:5.903937,Phylloscopus_borealis:5.903937):5.095898,(Phylloscopus_sibilatrix:10.113571,(Phylloscopus_trochilus:3.843395,Phylloscopus_collybita:3.843395):6.270176):0.886265):3.911051,Aegithalos_caudatus:14.910886):6.940866,((Delichon_urbicum:11.459644,Hirundo_rustica:11.459645):2.526993,Riparia_riparia:13.986637):7.865116):4.391475,(((((Acrocephalus_dumetorum:5.906465,Acrocephalus_scirpaceus:5.906465):3.581299,Acrocephalus_schoenobaenus:9.487763):0.574817,Acrocephalus_arundinaceus:10.062580):2.744321,Hippolais_icterina:12.806901):10.711527,((Locustella_naevia:7.072127,(Locustella_luscinioides:4.731653,Locustella_fluviatilis:4.731653):2.340473):8.862057,Acrocephalus_palustris:15.934183):7.584245):2.724800):2.080442):4.746860):3.961090):1.967279,(((Bombycilla_garrulus:35.930550,(((Troglodytes_troglodytes:22.682375,Certhia_familiaris:22.682375):2.857306,Sitta_europaea:25.539682):4.529923,(Sturnus_vulgaris:24.324066,(Cinclus_cinclus:23.783724,((Turdus_viscivorus:12.783412,(Turdus_philomelos:12.498227,((Turdus_merula:8.265793,Turdus_iliacus:8.265793):0.277432,(Turdus_torquatus:4.665226,Turdus_pilaris:4.665226):3.877999):3.955002):0.285185):9.299160,(Tarsiger_cyanurus:15.557327,(Muscicapa_striata:14.900421,((Erithacus_rubecula:13.655210,((Ficedula_hypoleuca:6.609611,Ficedula_parva:6.609611):6.767788,((Phoenicurus_ochruros:5.483768,Phoenicurus_phoenicurus:5.483768):7.046872,(Saxicola_rubetra:10.986797,Oenanthe_oenanthe:10.986797):1.543842):0.846760):0.277810):0.524039,(Luscinia_svecica:10.723803,Luscinia_luscinia:10.723803):3.455446):0.721171):0.656907):6.525244):1.701153):0.540342):5.745539):5.860947):1.647084,Regulus_regulus:37.577637):0.638386,(Prunella_modularis:31.255369,(((((Coccothraustes_coccothraustes:18.754223,((Carpodacus_erythrinus:14.445398,(Pyrrhula_pyrrhula:10.572211,Pinicola_enucleator:10.572211):3.873188):0.779330,(Carduelis_chloris:10.641123,((Serinus_serinus:7.991894,(Carduelis_cannabina:6.219900,Carduelis_spinus:6.219900):1.771995):0.069425,((Carduelis_flammea:5.323877,(Loxia_leucoptera:1.177438,(Loxia_pytyopsittacus:0.653326,Loxia_curvirostra:0.653326):0.524112):4.146439):1.725878,Carduelis_carduelis:7.049755):1.011565):2.579803):4.583606):3.529494):4.138854,(Fringilla_coelebs:7.025979,Fringilla_montifringilla:7.025979):15.867098):0.732029,(((Emberiza_hortulana:10.456762,Emberiza_citrinella:10.456762):3.342219,(Emberiza_schoeniclus:8.884407,((Emberiza_pusilla:4.779756,Emberiza_aureola:4.779756):1.715865,Emberiza_rustica:6.495620):2.388787):4.914573):7.171985,(Calcarius_lapponicus:18.510702,Plectrophenax_nivalis:18.510702):2.460264):2.654140):1.232091,((((Anthus_pratensis:4.283797,Anthus_petrosus:4.283797):2.966333,Anthus_cervinus:7.250130):4.529959,Anthus_trivialis:11.780089):9.013041,((Motacilla_flava:1.481362,Motacilla_citreola:1.481362):1.434544,(Motacilla_alba:2.770726,Motacilla_cinerea:2.770726):0.145180):17.877224):4.064067):1.128571,(Passer_montanus:5.575619,Passer_domesticus:5.575619):20.410149):5.269599):6.960655):0.782877):3.725303):34.938370,((((Falco_rusticolus:3.590094,Falco_peregrinus:3.590094):6.505319,Falco_tinnunculus:10.095412):0.233374,Falco_subbuteo:10.328786):1.538182,Falco_columbarius:11.866968):65.795601):2.150253,(Apus_apus:79.335587,(((Pernis_apivorus:41.876080,(Aquila_chrysaetos:30.960712,((((Circus_aeruginosus:13.075834,(Circus_cyaneus:9.772959,Circus_pygargus:9.772959):3.302877):8.528199,Accipiter_gentilis:21.604034):2.275930,Accipiter_nisus:23.879965):4.590845,((Milvus_migrans:19.125408,Haliaeetus_albicilla:19.125408):2.041309,(Buteo_lagopus:2.201516,Buteo_buteo:2.201516):18.965200):7.304091):2.489903):10.915368):20.374714,Pandion_haliaetus:62.250793):12.749772,(Caprimulgus_europaeus:74.834534,((((Glaucidium_passerinum:25.560698,Surnia_ulula:25.560698):7.142789,Aegolius_funereus:32.703487):7.559752,((Asio_flammeus:11.379297,Asio_otus:11.379296):18.721992,((Bubo_bubo:12.129286,Bubo_scandiaca:12.129286):9.974421,(Strix_nebulosa:11.558437,(Strix_aluco:8.694608,Strix_uralensis:8.694608):2.863829):10.545269):7.997583):10.161951):34.164379,((((Picoides_tridactylus:12.750525,(Dendrocopos_minor:9.922144,(Dendrocopos_leucotos:2.623832,Dendrocopos_major:2.623832):7.298311):2.828382):2.055577,(Picus_canus:10.948554,Dryocopus_martius:10.948554):3.857548):11.511502,Jynx_torquilla:26.317604):39.238384,Alcedo_atthis:65.555984):8.871634):0.406918):0.166027):4.335019):0.477241):1.546121):0.650065):3.867327):14.906076):0.238595;

TREE4=(((((Cygnus_cygnus:5.172361,Cygnus_olor:5.172361):3.842510,((Anser_fabalis:1.025784,Anser_anser:1.025784):4.968334,(Branta_leucopsis:1.369236,Branta_canadensis:1.369236):4.624882):3.020754):7.397509,((((Aythya_marila:0.920626,Aythya_fuligula:0.920626):0.781796,Aythya_ferina:1.702422):5.356447,(Tadorna_tadorna:6.990386,(((Melanitta_nigra:4.366442,Melanitta_fusca:4.366442):1.294406,(((Mergus_serrator:0.477066,Mergus_merganser:0.477066):3.351513,Mergellus_albellus:3.828579):1.405585,Bucephala_clangula:5.234164):0.426684):1.037333,(Somateria_mollissima:6.103891,Clangula_hyemalis:6.103891):0.594290):0.292204):0.068484):1.030855,((((Anas_acuta:2.531276,Anas_crecca:2.531276):0.066965,Anas_platyrhynchos:2.598240):1.441293,(Anas_strepera:1.879506,Anas_penelope:1.879506):2.160028):1.215207,(Anas_clypeata:2.549985,Anas_querquedula:2.549985):2.704755):2.834983):8.322656):60.062229,((Bonasa_bonasia:14.778123,((Tetrao_urogallus:4.876612,Tetrao_tetrix:4.876612):2.959658,(Lagopus_muta:3.227059,Lagopus_lagopus:3.227059):4.609211):6.941854):4.471372,(Phasianus_colchicus:16.906364,Perdix_perdix:16.906363):2.343132):57.225121):28.530945,(((Streptopelia_decaocto:9.470663,Streptopelia_turtur:9.470662):6.628586,(Columba_palumbus:9.705390,(Columba_oenas:6.404905,Columba_livia:6.404905):3.300484):6.393859):70.842697,((((Ardea_cinerea:43.213989,Botaurus_stellaris:43.213985):28.161722,(Phalacrocorax_carbo:70.180443,(Gavia_stellata:48.650040,Gavia_arctica:48.650040):21.530405):1.195264):10.710643,((((((Eudromias_morinellus:34.263844,(Charadrius_hiaticula:29.336473,Charadrius_dubius:29.336475):4.927371):16.379625,Vanellus_vanellus:50.643471):10.278601,Haematopus_ostralegus:60.922070):4.091327,Pluvialis_apricaria:65.013397):6.794528,(((Numenius_phaeopus:14.169802,Numenius_arquata:14.169802):29.394276,((Lymnocryptes_minimus:29.972334,(Limosa_lapponica:14.486004,Limosa_limosa:14.486004):15.486330):7.629539,((Arenaria_interpres:28.011532,((Philomachus_pugnax:18.145216,Limicola_falcinellus:18.145216):1.625905,(Calidris_alpina:19.080509,Calidris_temminckii:19.080509):0.690613):8.240410):5.238292,((Phalaropus_lobatus:31.323286,((Tringa_ochropus:19.848709,((Tringa_stagnatilis:8.348224,(Tringa_totanus:6.884694,Tringa_glareola:6.884694):1.463530):5.284706,(Tringa_erythropus:10.458447,Tringa_nebularia:10.458447):3.174483):6.215779):8.894843,Actitis_hypoleucos:28.743551):2.579732):1.657983,(Scolopax_rusticola:29.815351,(Gallinago_gallinago:12.164375,Gallinago_media:12.164375):17.650974):3.165916):0.268556):4.352050):5.962204):20.583294,(((Sterna_albifrons:13.220862,((Chlidonias_niger:8.050759,(Sterna_paradisaea:4.651484,Sterna_hirundo:4.651484):3.399275):1.559751,Sterna_caspia:9.610511):3.610352):8.680709,((Larus_ridibundus:4.585772,(Larus_canus:1.467562,((Larus_marinus:0.887159,Larus_argentatus:0.887159):0.116853,Larus_fuscus:1.004012):0.463550):3.118210):2.501120,Larus_minutus:7.086892):14.814679):3.196918,((Stercorarius_parasiticus:3.174213,Stercorarius_longicaudus:3.174213):20.005232,(Alca_torda:14.296206,Cepphus_grylle:14.296206):8.883240):1.919043):39.048882):7.660556):8.420968,((Tachybaptus_ruficollis:44.752552,(Podiceps_cristatus:11.975380,(Podiceps_auritus:8.081303,Podiceps_grisegena:8.081303):3.894076):32.777176):31.078644,(((((Gallinula_chloropus:13.363591,Fulica_atra:13.363591):16.939678,Porzana_porzana:30.303272):6.607175,Porzana_parva:36.910446):1.377503,(Crex_crex:17.756824,Rallus_aquaticus:17.756824):20.531124):24.203831,Grus_grus:62.491779):13.339421):4.397694):1.857460):2.083799,(Cuculus_canorus:83.789856,((((((Perisoreus_infaustus:19.951155,(Garrulus_glandarius:16.927404,((Nucifraga_caryocatactes:13.972252,((Corvus_corone:7.619376,(Corvus_frugilegus:5.933301,Corvus_corax:5.933301):1.686075):2.748354,Corvus_monedula:10.367730):3.604521):1.972278,Pica_pica:15.944530):0.982875):3.023750):7.164768,(Lanius_collurio:9.455183,Lanius_excubitor:9.455182):17.660740):4.759860,Oriolus_oriolus:31.875782):18.505684,((((Parus_major:18.368206,((Parus_ater:16.104025,(Parus_montanus:10.092030,Parus_cinctus:10.092030):6.011994):1.172546,Parus_cristatus:17.276569):1.091637):3.238158,Parus_caeruleus:21.606365):23.322935,((Panurus_biarmicus:26.470804,(Alauda_arvensis:10.675094,Lullula_arborea:10.675095):15.795708):12.281301,(((Aegithalos_caudatus:26.083004,(((Phylloscopus_collybita:5.584983,Phylloscopus_trochilus:5.584983):9.390443,Phylloscopus_sibilatrix:14.975427):2.058470,(Phylloscopus_borealis:11.897024,Phylloscopus_trochiloides:11.897025):5.136871):9.049107):6.759308,((Hirundo_rustica:16.835800,Delichon_urbicum:16.835798):2.236513,Riparia_riparia:19.072311):13.770000):2.282000,(((Acrocephalus_palustris:15.788696,((Locustella_luscinioides:4.186137,Locustella_fluviatilis:4.186137):4.673931,Locustella_naevia:8.860067):6.928628):7.288139,((((Acrocephalus_dumetorum:6.245175,Acrocephalus_scirpaceus:6.245176):2.312393,Acrocephalus_schoenobaenus:8.557569):0.431370,Acrocephalus_arundinaceus:8.988939):1.725662,Hippolais_icterina:10.714601):12.362234):1.703046,((Sylvia_communis:9.707726,(Sylvia_nisoria:8.475454,Sylvia_curruca:8.475455):1.232272):2.242615,(Sylvia_atricapilla:10.263710,Sylvia_borin:10.263710):1.686631):12.829540):10.344430):3.627795):6.177195):1.119522,((((((Certhia_familiaris:28.685593,Troglodytes_troglodytes:28.685593):3.535145,Sitta_europaea:32.220741):6.277349,(Sturnus_vulgaris:27.075211,(Cinclus_cinclus:26.714680,((Turdus_viscivorus:15.712634,(Turdus_philomelos:15.139011,((Turdus_iliacus:8.789974,Turdus_merula:8.789974):0.804810,(Turdus_pilaris:4.540750,Turdus_torquatus:4.540749):5.054035):5.544227):0.573624):8.909101,((Muscicapa_striata:16.216366,(Erithacus_rubecula:14.899773,(((Ficedula_parva:6.877974,Ficedula_hypoleuca:6.877973):7.115022,((Phoenicurus_ochruros:5.467503,Phoenicurus_phoenicurus:5.467503):7.074940,(Saxicola_rubetra:11.380982,Oenanthe_oenanthe:11.380983):1.161460):1.450552):0.825784,(Luscinia_svecica:11.447508,Luscinia_luscinia:11.447509):3.371271):0.080994):1.316592):0.071378,Tarsiger_cyanurus:16.287743):8.333992):2.092944):0.360531):11.422877):0.904077,Bombycilla_garrulus:39.402164):4.278518,Regulus_regulus:43.680679):0.105709,(Prunella_modularis:34.014782,(((((Coccothraustes_coccothraustes:19.893505,(((Pyrrhula_pyrrhula:13.524446,Pinicola_enucleator:13.524445):3.417304,Carpodacus_erythrinus:16.941750):0.743808,(((Carduelis_carduelis:9.994188,(Carduelis_flammea:6.984510,((Loxia_pytyopsittacus:0.735274,Loxia_curvirostra:0.735274):1.600417,Loxia_leucoptera:2.335691):4.648819):3.009678):0.668695,(Serinus_serinus:9.078370,(Carduelis_cannabina:7.351914,Carduelis_spinus:7.351914):1.726457):1.584512):1.681337,Carduelis_chloris:12.344219):5.341338):2.207949):8.499913,(Fringilla_coelebs:8.516909,Fringilla_montifringilla:8.516910):19.876511):0.748435,(((Emberiza_schoeniclus:5.032485,((Emberiza_pusilla:3.524794,Emberiza_aureola:3.524794):1.101112,Emberiza_rustica:4.625906):0.406580):4.145720,(Emberiza_hortulana:5.325611,Emberiza_citrinella:5.325611):3.852595):11.904579,(Calcarius_lapponicus:16.427446,Plectrophenax_nivalis:16.427446):4.655339):8.059070):1.378371,((Motacilla_cinerea:5.669389,(Motacilla_alba:4.987750,(Motacilla_citreola:4.189258,Motacilla_flava:4.189258):0.798492):0.681639):18.128534,((Anthus_cervinus:8.443701,(Anthus_petrosus:4.976505,Anthus_pratensis:4.976505):3.467196):3.759527,Anthus_trivialis:12.203228):11.594694):6.722302):0.847066,(Passer_montanus:5.486296,Passer_domesticus:5.486296):25.880995):2.647494):9.771606):2.262430):4.332644):28.945417,(((Falco_peregrinus:5.055324,Falco_rusticolus:5.055324):6.531404,(Falco_columbarius:8.664274,Falco_subbuteo:8.664274):2.922453):1.565894,Falco_tinnunculus:13.152621):66.174263):2.968622,(Apus_apus:81.684258,(((((((Strix_nebulosa:15.103334,(Strix_uralensis:11.911962,Strix_aluco:11.911962):3.191374):14.367591,(Bubo_bubo:14.138474,Bubo_scandiaca:14.138474):15.332451):5.689028,(Asio_otus:18.187660,Asio_flammeus:18.187662):16.972292):11.222430,((Surnia_ulula:25.286970,Glaucidium_passerinum:25.286972):9.105071,Aegolius_funereus:34.392044):11.990341):30.132343,((((Picus_canus:11.095167,Dryocopus_martius:11.095167):6.041705,((Dendrocopos_minor:10.571338,(Dendrocopos_major:2.250235,Dendrocopos_leucotos:2.250235):8.321103):3.189913,Picoides_tridactylus:13.761250):3.375621):12.722696,Jynx_torquilla:29.859568):39.205849,Alcedo_atthis:69.065422):7.449308):3.276572,Caprimulgus_europaeus:79.791290):0.096441,((Pernis_apivorus:42.080769,(Aquila_chrysaetos:31.125469,((Accipiter_nisus:23.377386,((Circus_pygargus:18.359993,(Circus_aeruginosus:11.316156,Circus_cyaneus:11.316156):7.043837):2.753705,Accipiter_gentilis:21.113697):2.263686):5.491040,((Buteo_lagopus:2.299124,Buteo_buteo:2.299124):21.721901,(Milvus_migrans:20.709827,Haliaeetus_albicilla:20.709827):3.311198):4.847399):2.257046):10.955296):16.767702,Pandion_haliaetus:58.848469):21.039274):1.796525):0.611240):1.494353):0.380293):2.771790):18.063612):12.175598;

TREE5=(((((Cygnus_cygnus:8.478786,Cygnus_olor:8.478786):4.592427,((Anser_anser:1.716976,Anser_fabalis:1.716976):7.006721,(Branta_leucopsis:2.886848,Branta_canadensis:2.886848):5.836848):4.347517):7.026828,(((((Somateria_mollissima:7.647206,((Bucephala_clangula:6.286457,((Mergus_merganser:1.115890,Mergus_serrator:1.115890):3.453456,Mergellus_albellus:4.569346):1.717111):0.581393,(Melanitta_fusca:5.020969,Melanitta_nigra:5.020969):1.846880):0.779357):0.349069,Clangula_hyemalis:7.996274):0.206278,Tadorna_tadorna:8.202554):0.633640,((Aythya_marila:1.166184,Aythya_fuligula:1.166184):0.439429,Aythya_ferina:1.605613):7.230580):0.612643,(((Anas_penelope:2.361363,Anas_strepera:2.361363):2.001855,(Anas_platyrhynchos:3.087149,(Anas_acuta:2.881611,Anas_crecca:2.881611):0.205538):1.276070):1.476240,(Anas_querquedula:2.913379,Anas_clypeata:2.913379):2.926080):3.609377):10.649204):56.532902,((Bonasa_bonasia:10.638586,((Tetrao_urogallus:4.632641,Tetrao_tetrix:4.632641):3.862823,(Lagopus_muta:3.466963,Lagopus_lagopus:3.466963):5.028501):2.143122):4.714001,(Phasianus_colchicus:14.444683,Perdix_perdix:14.444683):0.907902):61.278358):22.708143,(((Columba_palumbus:12.264752,(Columba_livia:8.126036,Columba_oenas:8.126036):4.138717):6.448176,(Streptopelia_turtur:10.644565,Streptopelia_decaocto:10.644565):8.068364):68.558617,(((((Botaurus_stellaris:45.237785,Ardea_cinerea:45.237782):24.536659,(Gavia_stellata:35.119385,Gavia_arctica:35.119385):34.655060):0.815857,Phalacrocorax_carbo:70.590302):6.729939,(((((Vanellus_vanellus:33.972027,((Charadrius_dubius:22.042831,Charadrius_hiaticula:22.042830):7.959352,Eudromias_morinellus:30.002182):3.969846):6.991833,Haematopus_ostralegus:40.963860):2.656272,Pluvialis_apricaria:43.620129):15.276421,(((Numenius_arquata:16.058641,Numenius_phaeopus:16.058641):19.840900,(((Arenaria_interpres:23.978636,((Calidris_temminckii:14.658637,Calidris_alpina:14.658638):2.196504,(Limicola_falcinellus:12.805537,Philomachus_pugnax:12.805537):4.049604):7.123494):4.549458,((((((Tringa_nebularia:10.762073,Tringa_erythropus:10.762073):2.562343,(Tringa_stagnatilis:6.429108,(Tringa_totanus:5.892889,Tringa_glareola:5.892889):0.536219):6.895308):4.041867,Tringa_ochropus:17.366283):6.187828,Actitis_hypoleucos:23.554111):0.256853,Phalaropus_lobatus:23.810963):3.782766,(Scolopax_rusticola:23.697105,(Gallinago_media:9.172930,Gallinago_gallinago:9.172930):14.524175):3.896626):0.934363):4.911979,(Lymnocryptes_minimus:25.715937,(Limosa_limosa:14.460518,Limosa_lapponica:14.460518):11.255420):7.724135):2.459468):18.597906,(((Larus_minutus:6.330546,(Larus_ridibundus:3.622697,(Larus_canus:1.165384,(Larus_fuscus:0.683544,(Larus_argentatus:0.524907,Larus_marinus:0.524907):0.158636):0.481841):2.457313):2.707849):11.449107,(Sterna_albifrons:11.265366,(((Sterna_hirundo:3.774860,Sterna_paradisaea:3.774860):3.986944,Chlidonias_niger:7.761804):1.059443,Sterna_caspia:8.821247):2.444119):6.514287):2.259854,((Stercorarius_longicaudus:5.268520,Stercorarius_parasiticus:5.268520):12.536910,(Alca_torda:14.458137,Cepphus_grylle:14.458138):3.347293):2.234077):34.457939):4.399106):13.982745,(((((Fulica_atra:16.386520,Gallinula_chloropus:16.386520):6.079038,(Porzana_porzana:17.685631,Porzana_parva:17.685629):4.779927):13.656393,(Rallus_aquaticus:14.055934,Crex_crex:14.055934):22.066017):20.097103,Grus_grus:56.219055):13.183841,(Tachybaptus_ruficollis:46.044819,((Podiceps_auritus:15.930300,Podiceps_cristatus:15.930299):0.931211,Podiceps_grisegena:16.861509):29.183308):23.358076):3.476403):4.440943):4.783309,(Cuculus_canorus:81.449707,(((Falco_tinnunculus:11.675517,(Falco_columbarius:10.735456,(Falco_subbuteo:9.464698,(Falco_rusticolus:3.648929,Falco_peregrinus:3.648929):5.815769):1.270758):0.940061):65.052124,((Oriolus_oriolus:22.216503,((((Pica_pica:10.478746,(Nucifraga_caryocatactes:8.098865,(Corvus_monedula:6.836617,(Corvus_corone:5.217686,(Corvus_frugilegus:4.842579,Corvus_corax:4.842578):0.375106):1.618932):1.262247):2.379883):0.590851,Garrulus_glandarius:11.069597):3.049692,Perisoreus_infaustus:14.119289):4.814178,(Lanius_excubitor:7.308577,Lanius_collurio:7.308576):11.624890):3.283036):28.057995,(((Parus_caeruleus:18.393787,(Parus_major:15.275486,(((Parus_montanus:9.595792,Parus_cinctus:9.595791):3.592151,Parus_cristatus:13.187943):0.552410,Parus_ater:13.740353):1.535133):3.118301):21.588253,((((((Delichon_urbicum:13.842247,Hirundo_rustica:13.842248):1.327375,Riparia_riparia:15.169621):11.970981,(Aegithalos_caudatus:21.936874,((Phylloscopus_sibilatrix:13.086706,(Phylloscopus_collybita:4.780394,Phylloscopus_trochilus:4.780394):8.306313):1.187995,(Phylloscopus_trochiloides:9.915215,Phylloscopus_borealis:9.915214):4.359488):7.662173):5.203729):3.145894,((Sylvia_borin:11.560315,Sylvia_atricapilla:11.560315):2.301701,(Sylvia_communis:11.404203,(Sylvia_nisoria:10.260479,Sylvia_curruca:10.260480):1.143725):2.457813):16.424480):1.053361,((((Locustella_fluviatilis:4.566059,Locustella_luscinioides:4.566059):4.236486,Locustella_naevia:8.802546):8.645882,Acrocephalus_palustris:17.448425):10.581141,(((Acrocephalus_schoenobaenus:10.034930,(Acrocephalus_scirpaceus:5.926844,Acrocephalus_dumetorum:5.926844):4.108087):0.733390,Acrocephalus_arundinaceus:10.768321):2.347733,Hippolais_icterina:13.116055):14.913512):3.310292):4.012776,(Panurus_biarmicus:31.412167,(Alauda_arvensis:12.626223,Lullula_arborea:12.626223):18.785944):3.940467):4.629406):4.251855,(((Bombycilla_garrulus:40.856838,((Sturnus_vulgaris:35.127659,(Cinclus_cinclus:32.003555,(((Turdus_merula:11.923356,(Turdus_iliacus:11.286522,(Turdus_pilaris:6.005976,Turdus_torquatus:6.005975):5.280546):0.636834):4.120505,(Turdus_philomelos:14.173912,Turdus_viscivorus:14.173912):1.869949):12.063592,(((((Luscinia_svecica:14.225885,Luscinia_luscinia:14.225885):2.385154,(((Oenanthe_oenanthe:12.884042,Saxicola_rubetra:12.884043):2.304591,(Phoenicurus_phoenicurus:5.944651,Phoenicurus_ochruros:5.944650):9.243983):0.711098,(Ficedula_hypoleuca:8.615947,Ficedula_parva:8.615947):7.283785):0.711308):0.732977,Erithacus_rubecula:17.344017):0.719711,Muscicapa_striata:18.063728):0.260521,Tarsiger_cyanurus:18.324247):9.783205):3.896102):3.124104):5.032219,((Certhia_familiaris:30.798918,Troglodytes_troglodytes:30.798918):4.146933,Sitta_europaea:34.945847):5.214030):0.696958):2.885487,Regulus_regulus:43.742321):0.053444,((((((Anthus_cervinus:7.238146,(Anthus_pratensis:4.414891,Anthus_petrosus:4.414892):2.823255):7.720031,Anthus_trivialis:14.958178):5.956826,(Motacilla_cinerea:4.404601,(Motacilla_alba:2.688780,(Motacilla_citreola:2.355619,Motacilla_flava:2.355619):0.333161):1.715821):16.510403):10.301001,(((Coccothraustes_coccothraustes:21.627043,((Carduelis_chloris:11.704522,((Carduelis_carduelis:9.524254,((Loxia_leucoptera:1.937743,(Loxia_pytyopsittacus:0.467445,Loxia_curvirostra:0.467445):1.470298):4.585971,Carduelis_flammea:6.523714):3.000539):0.111438,(Serinus_serinus:9.463568,(Carduelis_spinus:8.053327,Carduelis_cannabina:8.053326):1.410243):0.172123):2.068832):7.537619,(Carpodacus_erythrinus:18.562077,(Pinicola_enucleator:13.743141,Pyrrhula_pyrrhula:13.743140):4.818935):0.680067):2.384901):5.847081,(Fringilla_coelebs:9.033042,Fringilla_montifringilla:9.033042):18.441082):1.026766,((Calcarius_lapponicus:21.070770,Plectrophenax_nivalis:21.070770):1.750775,((Emberiza_hortulana:7.126110,Emberiza_citrinella:7.126110):5.034085,(Emberiza_schoeniclus:7.153880,((Emberiza_pusilla:4.545794,Emberiza_aureola:4.545794):0.935706,Emberiza_rustica:5.481500):1.672380):5.006314):10.661352):5.679344):2.715113):1.290981,(Passer_domesticus:8.438544,Passer_montanus:8.438544):24.068439):2.850243,Prunella_modularis:35.357231):8.438540):0.438127):6.040602):26.453146):2.972475,(Apus_apus:79.412338,((((((Glaucidium_passerinum:22.912624,Surnia_ulula:22.912624):3.793314,Aegolius_funereus:26.705938):8.070484,((((Strix_uralensis:5.962466,Strix_aluco:5.962465):4.405235,Strix_nebulosa:10.367701):11.673589,(Bubo_bubo:14.121061,Bubo_scandiaca:14.121061):7.920229):4.381638,(Asio_flammeus:9.346796,Asio_otus:9.346796):17.076132):8.353494):36.670120,((((Picus_canus:10.041414,Dryocopus_martius:10.041413):4.821781,((Dendrocopos_minor:8.921627,(Dendrocopos_major:2.117830,Dendrocopos_leucotos:2.117830):6.803796):3.154128,Picoides_tridactylus:12.075755):2.787440):11.060035,Jynx_torquilla:25.923229):39.295303,Alcedo_atthis:65.218529):6.228010):0.826576,Caprimulgus_europaeus:72.273117):1.888641,((Pernis_apivorus:39.513149,((((Milvus_migrans:19.785307,Haliaeetus_albicilla:19.785307):1.760658,(Buteo_lagopus:2.665384,Buteo_buteo:2.665384):18.880583):5.407335,((Accipiter_gentilis:19.005531,(Circus_cyaneus:9.932144,(Circus_pygargus:7.813364,Circus_aeruginosus:7.813363):2.118781):9.073385):0.402313,Accipiter_nisus:19.407845):7.545456):3.490726,Aquila_chrysaetos:30.444025):9.069124):19.893583,Pandion_haliaetus:59.406734):14.755024):5.250575):0.287785):1.749593):0.653839):5.167993):12.067544):8.985654;

TREE7=(((((Cygnus_cygnus:13.166034,Cygnus_olor:13.166033):4.624945,((Anser_anser:3.159656,Anser_fabalis:3.159656):8.227138,(Branta_canadensis:4.150697,Branta_leucopsis:4.150697):7.236096):6.404185):12.686461,(((Tadorna_tadorna:13.547256,((Aythya_marila:1.847387,Aythya_fuligula:1.847387):0.703103,Aythya_ferina:2.550489):10.996767):0.272767,((Clangula_hyemalis:11.266564,Somateria_mollissima:11.266564):1.473420,((Melanitta_nigra:9.058703,Melanitta_fusca:9.058702):1.971602,(((Mergus_serrator:1.344101,Mergus_merganser:1.344101):6.710814,Mergellus_albellus:8.054914):2.233901,Bucephala_clangula:10.288816):0.741489):1.709679):1.080039):2.522713,((((Anas_platyrhynchos:4.696509,Anas_crecca:4.696509):0.466834,Anas_acuta:5.163342):2.196661,(Anas_strepera:4.064374,Anas_penelope:4.064374):3.295629):3.554938,(Anas_clypeata:5.234318,Anas_querquedula:5.234318):5.680623):5.427795):14.134705):49.887520,((((Lagopus_muta:1.873477,Lagopus_lagopus:1.873477):3.434991,(Tetrao_urogallus:3.253352,Tetrao_tetrix:3.253352):2.055117):3.186145,Bonasa_bonasia:8.494613):4.512342,(Phasianus_colchicus:11.190710,Perdix_perdix:11.190709):1.816246):67.358002):28.796221,(((Streptopelia_decaocto:8.478607,Streptopelia_turtur:8.478607):5.687267,((Columba_livia:4.590806,Columba_oenas:4.590806):4.362405,Columba_palumbus:8.953212):5.212664):69.276123,((((Ardea_cinerea:42.064297,Botaurus_stellaris:42.064301):25.585331,(Phalacrocorax_carbo:65.314491,(Gavia_stellata:47.630505,Gavia_arctica:47.630505):17.683994):2.335130):6.944068,(((((Podiceps_grisegena:12.525377,Podiceps_auritus:12.525377):0.484423,Podiceps_cristatus:13.009800):10.753483,Tachybaptus_ruficollis:23.763283):43.735172,(((Porzana_parva:27.907017,((Fulica_atra:12.650353,Gallinula_chloropus:12.650354):4.916123,Porzana_porzana:17.566477):10.340540):4.830277,(Rallus_aquaticus:15.758242,Crex_crex:15.758242):16.979053):22.754097,Grus_grus:55.491386):12.007063):2.717650,((((Vanellus_vanellus:33.783455,((Charadrius_dubius:21.515663,Charadrius_hiaticula:21.515663):6.676366,Eudromias_morinellus:28.192028):5.591426):6.781775,Haematopus_ostralegus:40.565231):2.805715,Pluvialis_apricaria:43.370945):15.204700,(((Numenius_phaeopus:12.728510,Numenius_arquata:12.728510):19.371262,((Lymnocryptes_minimus:23.433620,(Limosa_limosa:12.688365,Limosa_lapponica:12.688365):10.745255):6.659374,(((Phalaropus_lobatus:25.247890,(Actitis_hypoleucos:23.575840,(((Tringa_nebularia:8.701748,Tringa_erythropus:8.701748):4.055779,((Tringa_stagnatilis:7.138733,Tringa_glareola:7.138733):1.428890,Tringa_totanus:8.567624):4.189904):2.110742,Tringa_ochropus:14.868270):8.707569):1.672051):3.065859,(Scolopax_rusticola:24.387438,(Gallinago_media:10.332685,Gallinago_gallinago:10.332685):14.054754):3.926310):0.881914,(Arenaria_interpres:23.671734,((Calidris_alpina:11.392660,Calidris_temminckii:11.392659):2.870099,(Philomachus_pugnax:12.846988,Limicola_falcinellus:12.846988):1.415770):9.408975):5.523930):0.897331):2.006776):15.239601,((((Sterna_caspia:6.580491,((Sterna_paradisaea:2.955476,Sterna_hirundo:2.955476):3.015487,Chlidonias_niger:5.970962):0.609528):5.214889,Sterna_albifrons:11.795380):6.155118,((Larus_ridibundus:3.638064,(Larus_canus:1.959314,(Larus_fuscus:0.668930,(Larus_argentatus:0.587635,Larus_marinus:0.587635):0.081296):1.290384):1.678749):1.860967,Larus_minutus:5.499031):12.451466):1.508500,((Alca_torda:13.159230,Cepphus_grylle:13.159230):3.594323,(Stercorarius_longicaudus:4.153702,Stercorarius_parasiticus:4.153702):12.599851):2.705445):27.880375):11.236272):11.640461):4.377593):8.352963,(Cuculus_canorus:82.842453,((((((Perisoreus_infaustus:16.998789,(((Nucifraga_caryocatactes:12.607641,(Corvus_monedula:9.288933,((Corvus_corax:5.701625,Corvus_frugilegus:5.701625):0.357992,Corvus_corone:6.059617):3.229316):3.318707):0.768994,Pica_pica:13.376635):0.923926,Garrulus_glandarius:14.300561):2.698227):7.502864,(Lanius_collurio:8.753212,Lanius_excubitor:8.753211):15.748440):4.065995,Oriolus_oriolus:28.567646):22.487890,(((Parus_caeruleus:20.159790,(Parus_major:17.319464,(Parus_ater:16.312733,((Parus_montanus:9.186773,Parus_cinctus:9.186773):6.493424,Parus_cristatus:15.680198):0.632535):1.006732):2.840326):20.687099,((Panurus_biarmicus:28.351635,(Lullula_arborea:10.147281,Alauda_arvensis:10.147281):18.204355):7.534249,(((Riparia_riparia:16.725376,(Hirundo_rustica:15.612797,Delichon_urbicum:15.612797):1.112579):14.119470,(Aegithalos_caudatus:27.051811,((Phylloscopus_borealis:11.993844,Phylloscopus_trochiloides:11.993845):7.877244,((Phylloscopus_trochilus:7.419972,Phylloscopus_collybita:7.419972):10.503976,Phylloscopus_sibilatrix:17.923948):1.947141):7.180723):3.793033):1.574391,((((Locustella_naevia:10.262794,(Locustella_fluviatilis:6.377860,Locustella_luscinioides:6.377860):3.884934):7.342367,Acrocephalus_palustris:17.605160):10.281545,(Hippolais_icterina:14.114656,((Acrocephalus_schoenobaenus:9.612749,(Acrocephalus_dumetorum:5.567701,Acrocephalus_scirpaceus:5.567701):4.045048):0.624590,Acrocephalus_arundinaceus:10.237340):3.877316):13.772049):3.207251,((Sylvia_atricapilla:8.692118,Sylvia_borin:8.692118):1.444977,((Sylvia_curruca:7.576054,Sylvia_nisoria:7.576053):0.481980,Sylvia_communis:8.058034):2.079062):20.956861):1.325280):3.466650):4.961004):3.026186,(((Bombycilla_garrulus:40.664314,(((Certhia_familiaris:27.150036,Troglodytes_troglodytes:27.150036):2.975645,Sitta_europaea:30.125681):8.587443,(Sturnus_vulgaris:25.771761,(Cinclus_cinclus:25.535824,((((Turdus_iliacus:8.463986,(Turdus_pilaris:4.989082,Turdus_torquatus:4.989082):3.474905):0.517390,Turdus_merula:8.981377):4.162998,(Turdus_philomelos:11.524683,Turdus_viscivorus:11.524683):1.619692):7.289677,(Tarsiger_cyanurus:14.232462,((Erithacus_rubecula:13.245990,((Luscinia_luscinia:8.877959,Luscinia_svecica:8.877959):3.861577,(((Oenanthe_oenanthe:9.921162,Saxicola_rubetra:9.921162):1.085480,(Phoenicurus_phoenicurus:4.804691,Phoenicurus_ochruros:4.804691):6.201951):1.177991,(Ficedula_parva:6.620911,Ficedula_hypoleuca:6.620911):5.563722):0.554902):0.506454):0.483982,Muscicapa_striata:13.729972):0.502489):6.201591):5.101772):0.235938):12.941363):1.951190):1.553381,Regulus_regulus:42.217697):0.833324,(Prunella_modularis:35.014400,((Passer_montanus:8.705219,Passer_domesticus:8.705218):22.035114,((((Coccothraustes_coccothraustes:18.777964,(((Pinicola_enucleator:11.450202,Pyrrhula_pyrrhula:11.450202):4.861485,Carpodacus_erythrinus:16.311687):0.661910,((((Carduelis_flammea:6.102290,((Loxia_pytyopsittacus:0.756569,Loxia_curvirostra:0.756569):1.369235,Loxia_leucoptera:2.125804):3.976486):2.930691,Carduelis_carduelis:9.032982):0.309538,((Carduelis_cannabina:6.145975,Carduelis_spinus:6.145976):3.094085,Serinus_serinus:9.240061):0.102459):2.644883,Carduelis_chloris:11.987403):4.986193):1.804368):6.013340,(Fringilla_coelebs:6.903248,Fringilla_montifringilla:6.903248):17.888056):1.322377,((Calcarius_lapponicus:16.529526,Plectrophenax_nivalis:16.529526):6.846838,((Emberiza_schoeniclus:5.136669,((Emberiza_pusilla:3.527765,Emberiza_aureola:3.527765):0.746458,Emberiza_rustica:4.274223):0.862446):3.245171,(Emberiza_hortulana:5.396987,Emberiza_citrinella:5.396987):2.984852):14.994525):2.737316):2.908378,((Anthus_trivialis:11.113020,(Anthus_cervinus:7.883734,(Anthus_petrosus:4.530422,Anthus_pratensis:4.530422):3.353312):3.229285):10.631858,(Motacilla_cinerea:5.323334,(Motacilla_alba:4.161942,(Motacilla_citreola:1.601673,Motacilla_flava:1.601673):2.560269):1.161392):16.421543):7.277181):1.718276):4.274064):8.036622):0.822056):7.182461):25.793631,(Falco_tinnunculus:13.402772,(((Falco_rusticolus:5.544255,Falco_peregrinus:5.544254):5.135868,Falco_subbuteo:10.680122):0.796562,Falco_columbarius:11.476686):1.926088):63.446396):4.864270,(Apus_apus:81.162651,(((Pernis_apivorus:40.450409,(Aquila_chrysaetos:32.491951,(((Milvus_migrans:18.761040,Haliaeetus_albicilla:18.761040):2.620792,(Buteo_lagopus:2.605960,Buteo_buteo:2.605960):18.775871):6.696163,((Accipiter_gentilis:21.573654,(Circus_cyaneus:9.600151,(Circus_pygargus:7.903002,Circus_aeruginosus:7.903001):1.697149):11.973503):1.324407,Accipiter_nisus:22.898060):5.179935):4.413956):7.958458):24.489086,Pandion_haliaetus:64.939491):12.879190,(Caprimulgus_europaeus:77.784927,((((((Strix_uralensis:8.326097,Strix_aluco:8.326097):2.396564,Strix_nebulosa:10.722660):16.316362,(Bubo_bubo:13.739904,Bubo_scandiaca:13.739904):13.299117):2.962155,(Asio_otus:14.618160,Asio_flammeus:14.618161):15.383017):13.731452,((Surnia_ulula:26.479868,Glaucidium_passerinum:26.479866):4.216363,Aegolius_funereus:30.696230):13.036398):33.246052,((((Picoides_tridactylus:7.727225,(Dendrocopos_minor:5.448565,(Dendrocopos_leucotos:1.396637,Dendrocopos_major:1.396637):4.051928):2.278661):1.471198,(Dryocopus_martius:6.383286,Picus_canus:6.383287):2.815136):6.709413,Jynx_torquilla:15.907835):50.120476,Alcedo_atthis:66.028305):10.950367):0.806246):0.033760):3.343963):0.550788):1.129021):0.104202):0.495341):25.719179):9.994455;

TREE8=(((((Cygnus_cygnus:7.331868,Cygnus_olor:7.331868):3.672397,((Anser_fabalis:1.716603,Anser_anser:1.716603):7.304538,(Branta_canadensis:1.962682,Branta_leucopsis:1.962682):7.058460):1.983123):13.404548,(((Anas_clypeata:3.055271,Anas_querquedula:3.055271):3.496372,(((Anas_acuta:3.906808,Anas_platyrhynchos:3.906808):0.313764,Anas_crecca:4.220572):1.080657,(Anas_penelope:2.961365,Anas_strepera:2.961365):2.339864):1.250414):4.032791,((Tadorna_tadorna:9.682121,((Aythya_marila:1.306315,Aythya_fuligula:1.306315):0.775767,Aythya_ferina:2.082083):7.600038):0.311364,(((((Mergus_merganser:0.782168,Mergus_serrator:0.782168):4.783435,Mergellus_albellus:5.565603):1.423235,Bucephala_clangula:6.988838):0.649680,(Melanitta_fusca:6.633293,Melanitta_nigra:6.633293):1.005225):1.108561,(Somateria_mollissima:7.954559,Clangula_hyemalis:7.954559):0.792521):1.246405):0.590949):13.824377):54.675354,((((Lagopus_muta:2.682943,Lagopus_lagopus:2.682943):6.050492,(Tetrao_tetrix:4.686747,Tetrao_urogallus:4.686747):4.046689):2.608353,Bonasa_bonasia:11.341789):5.318111,(Phasianus_colchicus:15.133752,Perdix_perdix:15.133753):1.526148):62.424271):31.142981,(((Columba_palumbus:12.727065,(Columba_oenas:8.432312,Columba_livia:8.432312):4.294753):9.303034,(Streptopelia_decaocto:10.466393,Streptopelia_turtur:10.466393):11.563705):65.647675,(((Phalacrocorax_carbo:72.024956,((Ardea_cinerea:46.788940,Botaurus_stellaris:46.788940):24.131920,(Gavia_stellata:42.165733,Gavia_arctica:42.165733):28.755125):1.104092):1.930880,((((Podiceps_grisegena:13.351677,(Podiceps_cristatus:11.168919,Podiceps_auritus:11.168919):2.182758):15.485399,Tachybaptus_ruficollis:28.837074):40.820335,(((Rallus_aquaticus:23.042345,Crex_crex:23.042347):16.393602,((Porzana_parva:14.553444,(Fulica_atra:13.779001,Gallinula_chloropus:13.779002):0.774443):13.809649,Porzana_porzana:28.363094):11.072855):11.369420,Grus_grus:50.805367):18.852045):2.671230,((((Vanellus_vanellus:35.075764,(Eudromias_morinellus:28.840425,(Charadrius_hiaticula:22.957472,Charadrius_dubius:22.957474):5.882953):6.235337):8.117058,Haematopus_ostralegus:43.192822):5.022777,Pluvialis_apricaria:48.215595):15.066343,(((Numenius_phaeopus:16.250013,Numenius_arquata:16.250013):23.426586,((Lymnocryptes_minimus:31.130808,(Limosa_limosa:11.749219,Limosa_lapponica:11.749219):19.381588):5.807250,(((Phalaropus_lobatus:29.064629,((Tringa_ochropus:21.853952,(((Tringa_glareola:6.269700,Tringa_totanus:6.269700):1.173941,Tringa_stagnatilis:7.443640):8.383255,(Tringa_erythropus:10.846185,Tringa_nebularia:10.846186):4.980710):6.027056):6.626801,Actitis_hypoleucos:28.480751):0.583876):3.088342,(Scolopax_rusticola:28.681347,(Gallinago_media:10.569822,Gallinago_gallinago:10.569822):18.111523):3.471626):0.984097,(Arenaria_interpres:26.361992,((Limicola_falcinellus:13.765829,Philomachus_pugnax:13.765829):3.510145,(Calidris_alpina:14.435133,Calidris_temminckii:14.435132):2.840841):9.086018):6.775076):3.800991):2.738542):16.654711,(((((Chlidonias_niger:8.533388,(Sterna_paradisaea:3.615177,Sterna_hirundo:3.615177):4.918211):2.473163,Sterna_caspia:11.006551):3.229951,Sterna_albifrons:14.236503):5.608309,((Larus_ridibundus:5.225052,(Larus_canus:1.587772,((Larus_argentatus:0.919317,Larus_fuscus:0.919317):0.092636,Larus_marinus:1.011953):0.575819):3.637279):0.885870,Larus_minutus:6.110922):13.733889):4.470114,((Alca_torda:14.058402,Cepphus_grylle:14.058401):6.710490,(Stercorarius_longicaudus:4.355282,Stercorarius_parasiticus:4.355282):16.413610):3.546033):32.016388):6.950627):9.046705):1.627186):10.165297,(Cuculus_canorus:83.593971,((((Oriolus_oriolus:26.761402,((Lanius_collurio:9.271548,Lanius_excubitor:9.271548):13.096778,((Garrulus_glandarius:13.799208,(Pica_pica:12.232809,(Nucifraga_caryocatactes:11.120405,(((Corvus_corax:6.115996,Corvus_frugilegus:6.115996):0.409220,Corvus_corone:6.525216):1.966306,Corvus_monedula:8.491522):2.628883):1.112404):1.566399):2.328401,Perisoreus_infaustus:16.127611):6.240717):4.393074):23.171318,((((Parus_major:16.441990,(Parus_cristatus:15.471700,(Parus_ater:15.319086,(Parus_montanus:10.093670,Parus_cinctus:10.093669):5.225417):0.152613):0.970292):3.017660,Parus_caeruleus:19.459652):25.126398,((Panurus_biarmicus:27.298138,(Lullula_arborea:10.160502,Alauda_arvensis:10.160502):17.137636):11.451742,(((Riparia_riparia:21.786861,(Delichon_urbicum:18.383808,Hirundo_rustica:18.383808):3.403054):7.108814,(((Locustella_naevia:10.877748,(Locustella_luscinioides:7.299148,Locustella_fluviatilis:7.299147):3.578600):9.244872,Acrocephalus_palustris:20.122620):7.019214,((Acrocephalus_arundinaceus:10.761874,(Acrocephalus_schoenobaenus:10.238958,(Acrocephalus_scirpaceus:6.058536,Acrocephalus_dumetorum:6.058536):4.180423):0.522916):2.900133,Hippolais_icterina:13.662007):13.479825):1.753844):7.217252,((Aegithalos_caudatus:27.517403,((Phylloscopus_borealis:13.097293,Phylloscopus_trochiloides:13.097293):6.232768,(Phylloscopus_sibilatrix:16.771196,(Phylloscopus_collybita:5.721864,Phylloscopus_trochilus:5.721864):11.049334):2.558865):8.187343):5.776002,((Sylvia_borin:12.123302,Sylvia_atricapilla:12.123302):2.529262,((Sylvia_curruca:10.066028,Sylvia_nisoria:10.066028):0.680030,Sylvia_communis:10.746058):3.906506):18.640842):2.819523):2.636952):5.836171):0.444320,(((((Sturnus_vulgaris:27.125671,(Cinclus_cinclus:26.558372,(((Turdus_philomelos:13.036858,((Turdus_torquatus:4.568641,Turdus_pilaris:4.568641):5.959140,(Turdus_iliacus:9.520099,Turdus_merula:9.520098):1.007683):2.509076):0.572354,Turdus_viscivorus:13.609212):12.183086,(Tarsiger_cyanurus:18.296415,(Muscicapa_striata:17.601263,(Erithacus_rubecula:16.436848,((((Phoenicurus_ochruros:5.168693,Phoenicurus_phoenicurus:5.168693):9.529616,(Saxicola_rubetra:11.896603,Oenanthe_oenanthe:11.896604):2.801705):0.324824,(Ficedula_parva:8.055914,Ficedula_hypoleuca:8.055914):6.967219):1.113767,(Luscinia_svecica:12.936832,Luscinia_luscinia:12.936832):3.200068):0.299947):1.164416):0.695154):7.495881):0.766076):0.567296):10.134360,((Troglodytes_troglodytes:25.538792,Certhia_familiaris:25.538794):3.940475,Sitta_europaea:29.479267):7.780762):5.486915,Bombycilla_garrulus:42.746941):1.046281,Regulus_regulus:43.793224):0.507318,((((((Coccothraustes_coccothraustes:20.108580,((Carpodacus_erythrinus:17.485928,(Pinicola_enucleator:12.145512,Pyrrhula_pyrrhula:12.145512):5.340416):0.106178,(Carduelis_chloris:10.950722,((Serinus_serinus:9.256058,(Carduelis_carduelis:7.585763,((Loxia_leucoptera:1.691582,(Loxia_pytyopsittacus:0.855888,Loxia_curvirostra:0.855888):0.835694):3.988254,Carduelis_flammea:5.679835):1.905928):1.670295):0.217834,(Carduelis_spinus:6.706616,Carduelis_cannabina:6.706615):2.767276):1.476830):6.641386):2.516474):8.815725,(Fringilla_coelebs:8.534065,Fringilla_montifringilla:8.534066):20.390242):1.197334,((((Emberiza_rustica:7.277228,(Emberiza_pusilla:4.737500,Emberiza_aureola:4.737500):2.539729):2.674790,Emberiza_schoeniclus:9.952019):4.099766,(Emberiza_citrinella:6.941461,Emberiza_hortulana:6.941461):7.110324):12.138485,(Calcarius_lapponicus:23.529531,Plectrophenax_nivalis:23.529530):2.660740):3.931370):1.524968,(((Anthus_cervinus:6.314075,(Anthus_pratensis:4.310007,Anthus_petrosus:4.310007):2.004069):3.536453,Anthus_trivialis:9.850529):13.019865,(Motacilla_cinerea:4.157538,((Motacilla_citreola:2.038073,Motacilla_flava:2.038073):1.443525,Motacilla_alba:3.481597):0.675941):18.712856):8.776216):1.855457,(Passer_domesticus:6.014386,Passer_montanus:6.014386):27.487680):3.131626,Prunella_modularis:36.633690):7.666852):0.729828):4.902348):30.878849,(Falco_tinnunculus:13.741042,((Falco_columbarius:8.785593,Falco_subbuteo:8.785593):1.445779,(Falco_rusticolus:3.301715,Falco_peregrinus:3.301715):6.929656):3.509672):67.070518):2.085289,(Apus_apus:82.614433,(((Pernis_apivorus:38.111298,(((((Circus_aeruginosus:11.671008,(Circus_pygargus:9.848441,Circus_cyaneus:9.848441):1.822567):9.879256,Accipiter_gentilis:21.550264):0.855914,Accipiter_nisus:22.406178):4.760811,((Milvus_migrans:19.667374,Haliaeetus_albicilla:19.667374):3.094022,(Buteo_lagopus:2.416892,Buteo_buteo:2.416892):20.344503):4.405594):3.435832,Aquila_chrysaetos:30.602821):7.508475):19.987219,Pandion_haliaetus:58.098515):21.109818,(Caprimulgus_europaeus:78.929947,((((Asio_flammeus:13.742729,Asio_otus:13.742729):17.507313,((Strix_nebulosa:10.288754,(Strix_aluco:7.106964,Strix_uralensis:7.106964):3.181790):11.414194,(Bubo_scandiaca:12.872006,Bubo_bubo:12.872007):8.830941):9.547094):7.349606,((Surnia_ulula:27.245462,Glaucidium_passerinum:27.245462):6.581483,Aegolius_funereus:33.826942):4.772701):37.542412,((((Picoides_tridactylus:8.090508,(Dendrocopos_minor:5.726394,(Dendrocopos_leucotos:1.284662,Dendrocopos_major:1.284662):4.441732):2.364112):1.726610,(Dryocopus_martius:6.934054,Picus_canus:6.934054):2.883062):6.651638,Jynx_torquilla:16.468754):55.085861,Alcedo_atthis:71.554619):4.587440):2.787895):0.278381):3.406105):0.282418):0.697111):0.527160):3.556644):22.549379):1.942332;

TREE9=(((((Cygnus_cygnus:6.645080,Cygnus_olor:6.645080):5.852900,((Anser_anser:0.980211,Anser_fabalis:0.980211):7.905105,(Branta_canadensis:1.424463,Branta_leucopsis:1.424463):7.460853):3.612664):7.015923,(((Tadorna_tadorna:8.900123,((Clangula_hyemalis:6.927114,Somateria_mollissima:6.927114):1.252626,(((Mergellus_albellus:5.929598,(Mergus_serrator:1.184185,Mergus_merganser:1.184185):4.745413):0.979887,Bucephala_clangula:6.909485):0.601903,(Melanitta_fusca:6.410252,Melanitta_nigra:6.410252):1.101137):0.668351):0.720383):0.671425,(Aythya_ferina:1.496090,(Aythya_marila:0.874219,Aythya_fuligula:0.874219):0.621872):8.075458):0.421366,((Anas_querquedula:3.861934,Anas_clypeata:3.861934):2.481399,(((Anas_acuta:3.567083,Anas_platyrhynchos:3.567083):0.269195,Anas_crecca:3.836277):1.146466,(Anas_strepera:2.633611,Anas_penelope:2.633611):2.349132):1.360590):3.649581):9.520988):54.965881,((Bonasa_bonasia:11.620790,((Tetrao_tetrix:3.989321,Tetrao_urogallus:3.989321):3.399631,(Lagopus_muta:3.117704,Lagopus_lagopus:3.117704):4.271248):4.231838):7.249697,(Phasianus_colchicus:17.236490,Perdix_perdix:17.236490):1.633996):55.609299):26.067162,((((Columba_livia:7.554918,Columba_oenas:7.554917):3.810437,Columba_palumbus:11.365355):9.000878,(Streptopelia_turtur:12.728135,Streptopelia_decaocto:12.728136):7.638096):68.362633,(((Phalacrocorax_carbo:73.404312,((Botaurus_stellaris:44.295456,Ardea_cinerea:44.295456):26.643240,(Gavia_stellata:35.891396,Gavia_arctica:35.891396):35.047298):2.465623):6.167109,(((Pluvialis_apricaria:47.511246,((Vanellus_vanellus:37.812847,((Charadrius_hiaticula:23.711653,Charadrius_dubius:23.711655):10.055205,Eudromias_morinellus:33.766857):4.045989):7.468525,Haematopus_ostralegus:45.281372):2.229872):15.090008,(((Numenius_arquata:11.829081,Numenius_phaeopus:11.829081):21.131918,((Lymnocryptes_minimus:24.139967,(Limosa_limosa:10.798985,Limosa_lapponica:10.798985):13.340981):4.816154,(((Gallinago_media:11.308916,Gallinago_gallinago:11.308916):12.168678,Scolopax_rusticola:23.477594):4.353419,((((Limicola_falcinellus:12.883136,Philomachus_pugnax:12.883136):1.458092,(Calidris_temminckii:11.946321,Calidris_alpina:11.946321):2.394906):6.933193,Arenaria_interpres:21.274420):5.668101,(Actitis_hypoleucos:23.451538,((Tringa_ochropus:17.161600,((Tringa_erythropus:10.242043,Tringa_nebularia:10.242043):1.268869,(Tringa_stagnatilis:6.253662,(Tringa_glareola:5.907455,Tringa_totanus:5.907455):0.346207):5.257249):5.650689):5.375100,Phalaropus_lobatus:22.536701):0.914838):3.490983):0.888491):1.125107):4.004879):18.852991,(((Sterna_albifrons:8.297557,((Chlidonias_niger:5.369923,(Sterna_paradisaea:2.713764,Sterna_hirundo:2.713764):2.656158):1.476743,Sterna_caspia:6.846665):1.450891):4.692846,(((Larus_canus:1.042022,(Larus_fuscus:0.894485,(Larus_argentatus:0.644117,Larus_marinus:0.644117):0.250368):0.147537):2.511517,Larus_ridibundus:3.553539):0.906327,Larus_minutus:4.459866):8.530538):1.516886,((Stercorarius_parasiticus:4.411256,Stercorarius_longicaudus:4.411256):8.950212,(Cepphus_grylle:10.656860,Alca_torda:10.656860):2.704608):1.145821):37.306702):10.787261):11.664090,(((((Porzana_porzana:10.472044,Porzana_parva:10.472045):4.348528,(Fulica_atra:6.722462,Gallinula_chloropus:6.722462):8.098111):5.040453,(Rallus_aquaticus:9.770681,Crex_crex:9.770681):10.090343):16.816965,Grus_grus:36.677990):35.347630,((Podiceps_cristatus:9.644688,(Podiceps_auritus:9.187059,Podiceps_grisegena:9.187059):0.457628):10.556118,Tachybaptus_ruficollis:20.200806):51.824814):2.239721):5.306084):6.777041,(Cuculus_canorus:85.984390,((((Falco_columbarius:10.054060,Falco_tinnunculus:10.054060):3.044099,(Falco_subbuteo:12.554968,(Falco_peregrinus:3.660537,Falco_rusticolus:3.660537):8.894431):0.543191):68.988617,((((Lanius_collurio:9.760185,Lanius_excubitor:9.760185):17.391893,((Garrulus_glandarius:17.279793,((Nucifraga_caryocatactes:13.022133,(Corvus_monedula:9.374042,((Corvus_frugilegus:6.422220,Corvus_corax:6.422220):0.781658,Corvus_corone:7.203877):2.170164):3.648091):2.190521,Pica_pica:15.212654):2.067139):2.227914,Perisoreus_infaustus:19.507708):7.644372):3.492071,Oriolus_oriolus:30.644148):23.506918,(((Parus_caeruleus:19.845287,(Parus_major:16.921583,((Parus_montanus:9.580564,Parus_cinctus:9.580564):5.449463,(Parus_cristatus:14.642331,Parus_ater:14.642330):0.387696):1.891555):2.923706):25.541775,((((Hirundo_rustica:18.808632,Delichon_urbicum:18.808632):2.670401,Riparia_riparia:21.479033):16.133568,((((Sylvia_atricapilla:8.665664,Sylvia_borin:8.665665):4.659232,((Sylvia_nisoria:11.031430,Sylvia_curruca:11.031431):0.212284,Sylvia_communis:11.243713):2.081182):15.877151,(Aegithalos_caudatus:27.293154,((Phylloscopus_sibilatrix:17.860474,(Phylloscopus_trochilus:6.877111,Phylloscopus_collybita:6.877111):10.983362):2.512817,(Phylloscopus_borealis:10.979359,Phylloscopus_trochiloides:10.979359):9.393932):6.919863):1.908894):8.219899,((Hippolais_icterina:17.167377,(Acrocephalus_arundinaceus:12.801009,(Acrocephalus_schoenobaenus:12.276838,(Acrocephalus_dumetorum:6.978237,Acrocephalus_scirpaceus:6.978237):5.298602):0.524171):4.366367):15.984662,((Locustella_naevia:11.347902,(Locustella_luscinioides:6.265886,Locustella_fluviatilis:6.265885):5.082017):9.882041,Acrocephalus_palustris:21.229944):11.922094):4.269907):0.190655):3.481182,(Panurus_biarmicus:34.459263,(Alauda_arvensis:11.910160,Lullula_arborea:11.910159):22.549103):6.634521):4.293278):2.268208,((Regulus_regulus:46.813236,((((Certhia_familiaris:31.413694,Troglodytes_troglodytes:31.413692):2.966231,Sitta_europaea:34.379925):8.612809,(Sturnus_vulgaris:31.910028,(Cinclus_cinclus:30.123312,((((Turdus_pilaris:5.516744,Turdus_torquatus:5.516744):4.581515,(Turdus_iliacus:9.747904,Turdus_merula:9.747903):0.350356):4.618298,(Turdus_viscivorus:13.327669,Turdus_philomelos:13.327669):1.388888):12.947397,(Tarsiger_cyanurus:19.399248,((Erithacus_rubecula:18.799593,((((Phoenicurus_phoenicurus:7.597809,Phoenicurus_ochruros:7.597809):8.557296,(Saxicola_rubetra:14.897777,Oenanthe_oenanthe:14.897777):1.257329):1.033678,(Ficedula_parva:10.718607,Ficedula_hypoleuca:10.718607):6.470176):1.296466,(Luscinia_svecica:14.718540,Luscinia_luscinia:14.718539):3.766709):0.314344):0.598013,Muscicapa_striata:19.397606):0.001643):8.264706):2.459357):1.786717):11.082706):2.423498,Bombycilla_garrulus:45.416233):1.397007):0.218327,(Prunella_modularis:35.869122,(((Anthus_trivialis:12.596260,((Anthus_petrosus:2.309356,Anthus_pratensis:2.309356):5.318991,Anthus_cervinus:7.628346):4.967913):8.802278,(Motacilla_cinerea:4.861551,((Motacilla_citreola:3.165218,Motacilla_flava:3.165218):0.889068,Motacilla_alba:4.054286):0.807265):16.536987):11.656770,((Passer_domesticus:6.704902,Passer_montanus:6.704902):25.025572,(((Coccothraustes_coccothraustes:21.572578,(((Carduelis_carduelis:9.608740,(((Loxia_leucoptera:2.680825,(Loxia_pytyopsittacus:0.447753,Loxia_curvirostra:0.447753):2.233072):4.836673,Carduelis_flammea:7.517498):1.919229,(Serinus_serinus:8.891770,(Carduelis_cannabina:7.239207,Carduelis_spinus:7.239207):1.652564):0.544957):0.172013):3.477726,Carduelis_chloris:13.086466):6.592345,((Pyrrhula_pyrrhula:14.176754,Pinicola_enucleator:14.176752):4.087644,Carpodacus_erythrinus:18.264399):1.414415):1.893768):7.074755,(Fringilla_coelebs:9.807929,Fringilla_montifringilla:9.807929):18.839405):0.497130,((Calcarius_lapponicus:19.946583,Plectrophenax_nivalis:19.946583):6.808884,((Emberiza_schoeniclus:10.478697,((Emberiza_pusilla:6.158598,Emberiza_aureola:6.158598):2.489563,Emberiza_rustica:8.648161):1.830535):5.552741,(Emberiza_citrinella:10.237968,Emberiza_hortulana:10.237969):5.793468):10.724029):2.388998):2.586010):1.324834):2.813816):11.162443):0.623703):6.495799):27.935707):3.084124,(Apus_apus:85.151062,(((Pernis_apivorus:45.756393,(Aquila_chrysaetos:36.608521,(((Milvus_migrans:24.695080,Haliaeetus_albicilla:24.695080):3.142787,(Buteo_buteo:3.226797,Buteo_lagopus:3.226797):24.611069):4.915491,(((Circus_aeruginosus:9.639787,(Circus_cyaneus:0.947035,Circus_pygargus:0.947035):8.692751):11.251823,Accipiter_gentilis:20.891611):3.797515,Accipiter_nisus:24.689125):8.064231):3.855165):9.147876):19.446194,Pandion_haliaetus:65.202591):16.621006,(((((Glaucidium_passerinum:28.880617,Surnia_ulula:28.880619):5.615499,Aegolius_funereus:34.496117):10.337238,(((Bubo_bubo:12.863127,Bubo_scandiaca:12.863128):11.372694,((Strix_aluco:7.607452,Strix_uralensis:7.607452):3.111573,Strix_nebulosa:10.719025):13.516797):8.927902,(Asio_flammeus:13.788727,Asio_otus:13.788729):19.374996):11.669632):35.096836,((((Dryocopus_martius:11.769480,Picus_canus:11.769482):4.706942,(((Dendrocopos_major:3.133420,Dendrocopos_leucotos:3.133420):7.357528,Dendrocopos_minor:10.490948):3.348572,Picoides_tridactylus:13.839520):2.636904):11.981199,Jynx_torquilla:28.457623):42.391792,Alcedo_atthis:70.849411):9.080782):1.658595,Caprimulgus_europaeus:81.588791):0.234806):3.327458):0.019846):0.813502):0.364066):2.380398):11.818082):2.695615;

TREE10=(((((Cygnus_olor:12.169790,Cygnus_cygnus:12.169790):5.651125,((Anser_anser:2.284276,Anser_fabalis:2.284276):9.718407,(Branta_canadensis:1.750285,Branta_leucopsis:1.750285):10.252398):5.818233):10.487334,(((Anas_clypeata:4.119473,Anas_querquedula:4.119473):3.909000,((Anas_penelope:2.778442,Anas_strepera:2.778442):3.554135,((Anas_acuta:4.789163,Anas_crecca:4.789163):0.211048,Anas_platyrhynchos:5.000211):1.332366):1.695896):5.434794,((Aythya_ferina:2.363123,(Aythya_marila:1.717866,Aythya_fuligula:1.717866):0.645257):10.601280,(Tadorna_tadorna:12.219035,(Clangula_hyemalis:11.310486,(Somateria_mollissima:10.475146,((Melanitta_fusca:7.421308,Melanitta_nigra:7.421309):2.797273,(((Mergus_merganser:1.361868,Mergus_serrator:1.361868):6.033824,Mergellus_albellus:7.395692):2.240914,Bucephala_clangula:9.636606):0.581975):0.256565):0.835339):0.908549):0.745369):0.498863):14.844984):55.779465,((Bonasa_bonasia:6.778716,((Tetrao_tetrix:2.622390,Tetrao_urogallus:2.622390):1.926450,(Lagopus_muta:2.100229,Lagopus_lagopus:2.100229):2.448611):2.229875):3.943413,(Phasianus_colchicus:8.716753,Perdix_perdix:8.716753):2.005375):73.365593):22.044283,(((Columba_palumbus:11.445711,(Columba_oenas:7.365536,Columba_livia:7.365536):4.080176):9.070053,(Streptopelia_decaocto:10.827978,Streptopelia_turtur:10.827978):9.687786):67.032654,((((Ardea_cinerea:44.180191,Botaurus_stellaris:44.180191):27.701328,(Phalacrocorax_carbo:68.180962,(Gavia_stellata:35.486958,Gavia_arctica:35.486958):32.694000):3.700562):2.062873,(((((((Charadrius_hiaticula:24.640308,Charadrius_dubius:24.640310):4.789186,Eudromias_morinellus:29.429497):8.631433,Vanellus_vanellus:38.060928):2.695111,Haematopus_ostralegus:40.756042):0.873569,Pluvialis_apricaria:41.629612):18.571968,(((Numenius_phaeopus:13.612880,Numenius_arquata:13.612881):23.668236,(((Arenaria_interpres:28.514280,((Philomachus_pugnax:18.242947,Limicola_falcinellus:18.242947):0.924512,(Calidris_temminckii:13.190138,Calidris_alpina:13.190140):5.977318):9.346823):1.210988,((((Tringa_ochropus:21.274696,((Tringa_nebularia:10.557755,Tringa_erythropus:10.557755):5.121541,(Tringa_stagnatilis:6.454410,(Tringa_totanus:6.092211,Tringa_glareola:6.092211):0.362199):9.224887):5.595401):7.076488,Actitis_hypoleucos:28.351185):0.095802,Phalaropus_lobatus:28.446987):0.678172,(Scolopax_rusticola:23.573816,(Gallinago_gallinago:9.848105,Gallinago_media:9.848105):13.725710):5.551344):0.600109):4.508524,(Lymnocryptes_minimus:30.571005,(Limosa_lapponica:14.557858,Limosa_limosa:14.557858):16.013147):3.662789):3.047322):14.815625,((((Sterna_caspia:8.281794,(Chlidonias_niger:7.245571,(Sterna_hirundo:3.028515,Sterna_paradisaea:3.028515):4.217056):1.036223):2.695787,Sterna_albifrons:10.977582):7.181385,((Larus_ridibundus:4.108943,(((Larus_argentatus:0.774373,Larus_marinus:0.774373):0.098005,Larus_fuscus:0.872378):0.548524,Larus_canus:1.420902):2.688040):0.712983,Larus_minutus:4.821926):13.337040):1.961749,((Alca_torda:12.312423,Cepphus_grylle:12.312423):5.941239,(Stercorarius_longicaudus:3.850467,Stercorarius_parasiticus:3.850467):14.403194):1.867053):31.976025):8.104840):10.314923,((((Podiceps_auritus:13.930519,Podiceps_cristatus:13.930519):4.646469,Podiceps_grisegena:18.576988):17.670975,Tachybaptus_ruficollis:36.247963):32.833496,((((Fulica_atra:14.201138,Gallinula_chloropus:14.201136):5.900652,(Porzana_parva:13.476974,Porzana_porzana:13.476975):6.624813):10.495043,(Rallus_aquaticus:16.070055,Crex_crex:16.070055):14.526776):16.611780,Grus_grus:47.208611):21.872849):1.435044):3.427887):9.494941,(Cuculus_canorus:82.969193,((((((Perisoreus_infaustus:13.850071,((Nucifraga_caryocatactes:10.418280,(((Corvus_frugilegus:4.545604,Corvus_corax:4.545604):1.701136,Corvus_corone:6.246739):2.152078,Corvus_monedula:8.398818):2.019462):1.288490,(Pica_pica:11.151598,Garrulus_glandarius:11.151598):0.555171):2.143301):4.954634,(Lanius_excubitor:7.904841,Lanius_collurio:7.904840):10.899864):3.366700,Oriolus_oriolus:22.171402):27.402958,(((Parus_caeruleus:22.004190,(Parus_major:17.928659,(Parus_ater:15.848166,(Parus_cristatus:15.391483,(Parus_montanus:8.981837,Parus_cinctus:8.981837):6.409646):0.456683):2.080492):4.075531):17.725494,((Panurus_biarmicus:29.058664,(Alauda_arvensis:12.428996,Lullula_arborea:12.428996):16.629667):4.730306,((((Sylvia_curruca:10.866279,Sylvia_nisoria:10.866278):0.320190,Sylvia_communis:11.186468):5.091939,(Sylvia_atricapilla:12.972507,Sylvia_borin:12.972507):3.305900):14.963715,(((((Locustella_luscinioides:6.687443,Locustella_fluviatilis:6.687443):4.359574,Locustella_naevia:11.047017):9.397124,Acrocephalus_palustris:20.444141):9.210802,(Hippolais_icterina:14.233945,((Acrocephalus_schoenobaenus:10.425342,(Acrocephalus_dumetorum:6.505138,Acrocephalus_scirpaceus:6.505138):3.920203):0.650777,Acrocephalus_arundinaceus:11.076118):3.157826):15.420998):0.994769,((Aegithalos_caudatus:22.772041,(((Phylloscopus_trochilus:5.536466,Phylloscopus_collybita:5.536466):8.551600,Phylloscopus_sibilatrix:14.088066):1.480553,(Phylloscopus_trochiloides:10.724402,Phylloscopus_borealis:10.724402):4.844216):7.203424):4.685910,((Delichon_urbicum:13.796389,Hirundo_rustica:13.796388):1.987524,Riparia_riparia:15.783913):11.674040):3.191761):0.592410):2.546847):5.940713):4.419631,(((Bombycilla_garrulus:41.424938,(((Troglodytes_troglodytes:26.412434,Certhia_familiaris:26.412434):5.289570,Sitta_europaea:31.702003):7.139260,(Sturnus_vulgaris:24.259899,(Cinclus_cinclus:21.606180,(((Turdus_viscivorus:9.798696,Turdus_philomelos:9.798696):0.254968,((Turdus_iliacus:6.429487,Turdus_merula:6.429486):0.522296,(Turdus_torquatus:3.552938,Turdus_pilaris:3.552938):3.398845):3.101881):9.987690,(((((((Phoenicurus_phoenicurus:5.019036,Phoenicurus_ochruros:5.019036):5.986366,(Saxicola_rubetra:9.855692,Oenanthe_oenanthe:9.855692):1.149710):1.019670,(Ficedula_parva:5.886993,Ficedula_hypoleuca:5.886993):6.138079):0.554782,(Luscinia_luscinia:9.961477,Luscinia_svecica:9.961477):2.618377):0.608045,Erithacus_rubecula:13.187899):0.723627,Muscicapa_striata:13.911526):0.006392,Tarsiger_cyanurus:13.917917):6.123436):1.564827):2.653719):14.581365):2.583673):1.773853,Regulus_regulus:43.198788):0.116248,(Prunella_modularis:36.586540,((((Anthus_trivialis:9.071228,(Anthus_cervinus:5.106968,(Anthus_petrosus:3.861228,Anthus_pratensis:3.861228):1.245740):3.964260):15.151535,(Motacilla_cinerea:4.318784,((Motacilla_flava:2.401371,Motacilla_citreola:2.401371):0.457141,Motacilla_alba:2.858512):1.460272):19.903980):7.870635,(((Coccothraustes_coccothraustes:22.071613,((Pyrrhula_pyrrhula:13.017452,Pinicola_enucleator:13.017453):5.727721,(Carpodacus_erythrinus:18.322136,(Carduelis_chloris:12.222991,((Carduelis_carduelis:9.267829,((Loxia_leucoptera:1.101915,(Loxia_pytyopsittacus:0.462295,Loxia_curvirostra:0.462295):0.639620):5.438216,Carduelis_flammea:6.540132):2.727697):0.820024,((Carduelis_cannabina:8.023790,Carduelis_spinus:8.023790):1.814236,Serinus_serinus:9.838026):0.249826):2.135139):6.099144):0.423039):3.326440):6.378177,(Fringilla_coelebs:7.075154,Fringilla_montifringilla:7.075154):21.374636):2.278786,((Calcarius_lapponicus:18.407021,Plectrophenax_nivalis:18.407021):8.387632,((((Emberiza_pusilla:5.484123,Emberiza_aureola:5.484123):2.078738,Emberiza_rustica:7.562861):2.435276,Emberiza_schoeniclus:9.998137):3.364056,(Emberiza_citrinella:10.272318,Emberiza_hortulana:10.272318):3.089874):13.432461):3.933923):1.364822):1.472661,(Passer_montanus:5.855045,Passer_domesticus:5.855045):27.711016):3.020481):6.728498):0.834276):5.425048):29.804743,((Falco_subbuteo:10.289648,(Falco_peregrinus:2.885663,Falco_rusticolus:2.885663):7.403985):1.054709,(Falco_tinnunculus:10.851835,Falco_columbarius:10.851835):0.492521):68.034744):1.071576,(Apus_apus:79.935822,((Caprimulgus_europaeus:77.845413,((((Asio_otus:10.814173,Asio_flammeus:10.814172):18.340416,((Strix_nebulosa:10.582880,(Strix_aluco:8.473258,Strix_uralensis:8.473258):2.109622):13.338625,(Bubo_bubo:11.709341,Bubo_scandiaca:11.709341):12.212166):5.233082):11.158419,((Surnia_ulula:24.991032,Glaucidium_passerinum:24.991032):2.946306,Aegolius_funereus:27.937336):12.375669):36.208576,((((Picoides_tridactylus:16.514275,(Dendrocopos_minor:13.036222,(Dendrocopos_leucotos:3.618720,Dendrocopos_major:3.618719):9.417501):3.478053):3.196998,(Picus_canus:14.107327,Dryocopus_martius:14.107328):5.603944):14.419441,Jynx_torquilla:34.130714):36.347366,Alcedo_atthis:70.478073):6.043508):1.323825):0.021073,((Pernis_apivorus:41.564098,(Aquila_chrysaetos:31.800648,((((Circus_cyaneus:10.933285,(Circus_pygargus:6.723802,Circus_aeruginosus:6.723802):4.209484):10.479347,Accipiter_gentilis:21.412632):1.610268,Accipiter_nisus:23.022900):4.874510,((Buteo_lagopus:2.428988,Buteo_buteo:2.428988):20.333500,(Milvus_migrans:20.812286,Haliaeetus_albicilla:20.812286):1.950202):5.134923):3.903237):9.763452):19.421452,Pandion_haliaetus:60.985550):16.880932):2.069338):0.514860):2.518509):0.470141):4.109093):18.583576):6.334105;
